# Measles skin rash: Infection of lymphoid and myeloid cells in the dermis precedes viral dissemination to the epidermis

**DOI:** 10.1371/journal.ppat.1008253

**Published:** 2020-10-08

**Authors:** Brigitta M. Laksono, Paola Fortugno, Bernadien M. Nijmeijer, Rory D. de Vries, Sonia Cordisco, Thijs Kuiken, Teunis B. H. Geijtenbeek, W. Paul Duprex, Francesco Brancati, Rik L. de Swart

**Affiliations:** 1 Department of Viroscience, Erasmus MC, University Medical Center Rotterdam, Rotterdam, the Netherlands; 2 Laboratory of Molecular and Cell Biology, Istituto Dermopatico dell'Immacolata, IDI-IRCCS, Rome, Italy; 3 Department of Experimental Immunology, Amsterdam Infection and Immunity Institute, Amsterdam University Medical Centers, University of Amsterdam, Amsterdam, the Netherlands; 4 Centre for Vaccine Research, University of Pittsburgh School of Medicine, Pennsylvania, United States of America; Boston University, UNITED STATES

## Abstract

Measles is characterized by fever and a maculopapular skin rash, which is accompanied by immune clearance of measles virus (MV)-infected cells. Histopathological analyses of skin biopsies from humans and non-human primates (NHPs) with measles rash have identified MV-infected keratinocytes and mononuclear cells in the epidermis, around hair follicles and near sebaceous glands. Here, we address the pathogenesis of measles skin rash by combining data from experimentally infected NHPs, *ex vivo* infection of human skin sheets and *in vitro* infection of primary human keratinocytes. Analysis of NHP skin samples collected at different time points following MV inoculation demonstrated that infection in the skin precedes onset of rash by several days. MV infection was detected in lymphoid and myeloid cells in the dermis before dissemination to the epidermal leukocytes and keratinocytes. These data were in good concordance with *ex vivo* MV infections of human skin sheets, in which dermal cells were more targeted than the epidermal cells. To address viral dissemination to the epidermis and to determine whether the dissemination is receptor-dependent, we performed experimental infections of primary keratinocytes collected from healthy donors. These experiments demonstrated that MV infection of keratinocytes is mainly nectin-4-dependent, and differentiated keratinocytes, which express higher levels of nectin-4, are more susceptible to MV infection than proliferating keratinocytes. Based on these data, we propose a model to explain measles skin rash: migrating MV-infected lymphocytes initiate the infection of dermal skin-resident CD150^+^ immune cells. The infection is subsequently disseminated from the dermal papillae to nectin-4^+^ keratinocytes in the basal epidermis. Lateral spread of MV infection is observed in the superficial epidermis, most likely due to the higher level of nectin-4 expression on differentiated keratinocytes. Finally, MV-infected cells are cleared by infiltrating immune cells, causing hyperemia and edema, which give the appearance of morbilliform skin rash.

## Author summary

Several viral infections are associated with skin rash, including parvovirus B19, human herpesvirus type 6, dengue virus and rubella virus. However, the archetype virus infection that leads to skin rash is measles. Although all of these viral exanthemata often appear similar, their pathogenesis is different. In the case of measles, the appearance of skin rash is a sign that the immune system is clearing MV-infected cells from the skin. How the virus reaches the skin and is locally disseminated remains unknown. Here, we combine observations and expertise from pathologists, dermatologists, virologists and immunologists to delineate the pathogenesis of measles skin rash. We show that MV infection of dermal myeloid and lymphoid cells precedes viral dissemination to the epidermal leukocytes and keratinocytes. We speculate that immune-mediated clearance of these infected cells results in hyperemia and edema, explaining the redness of the skin and the slightly elevated spots of the morbilliform rash.

## Introduction

Measles virus (MV) is a highly contagious enveloped virus with a negative single-stranded RNA genome that belongs to the family *Paramyxoviridae*, genus *Morbillivirus* [1]. Measles is associated with fever, cough and a characteristic maculopapular skin rash [2]. MV utilizes two cellular receptors to infect its target cells: CD150 and nectin-4 [3–5]. CD150 plays a crucial role during viral entry and systemic dissemination. It is expressed on subsets of immune cells, including macrophages, dendritic cells (DCs) and lymphocytes. Nectin-4 is crucial for viral transmission to the next host. It is an adherens junction protein expressed at the basolateral surface of differentiated respiratory epithelial cells and is involved in the maintenance of epithelial integrity [6, 7].

Following entry of MV into the respiratory tract, the primary infection of myeloid cells leads to a cell-associated viremia mediated by CD150^+^ lymphocytes, resulting in systemic disease [8–10]. During a clinically silent incubation phase of 7 to 10 days, circulating MV-infected lymphocytes migrate into various tissues and transmit the virus to susceptible tissue-resident CD150^+^ immune cells and nectin-4^+^ epithelial cells. Basolateral infection of respiratory epithelial cells leads to the apical release of nascent virions into the lumen of the respiratory tract [11–13]. Shedding is associated with the onset of prodromal clinical signs such as fever and cough [2, 10]. Maculopapular skin rash and conjunctivitis follow a few days later [10] and are associated with onset of MV-specific cellular immune responses [2]. Patients with a compromised cellular immune system do not develop rash or conjunctivitis, but are at high risk of developing severe disease [14].

In histopathological studies of human skin biopsies, measles skin rash is mostly characterized by infection and necrosis of keratinocytes and mononuclear cells in the epidermis, and multinucleated giant cells located in proximity to hair follicles and sebaceous glands [15, 16]. It has been postulated that measles rash starts by infection of dermal endothelial cells [17]. However, these cells neither express CD150 nor nectin-4 [18, 19]. Moreover, we have previously identified MV-infected lymphocytes and DCs in the skin of experimentally infected non-human primates preceding onset of skin rash [9]. Besides CD150^+^ and nectin-4^+^ cells, other cells that express DC-SIGN or Langerin could play a role in the pathogenesis of measles skin rash, since DC-SIGN and Langerin facilitate attachment, but not entry, of MV and thus potentially help in spreading the infection in the skin [20, 21].

In order to understand the pathogenesis of measles skin rash, it is important to understand both the architecture of the skin and the spatial organization of cell subsets that express either CD150 or nectin-4. The dermis is vascularized and contains several subsets of immune cells that express CD150. These include a network of myeloid DCs and clusters of tissue-resident CD4^+^ and CD8^+^ T cells [22–24]. In contrast to the dermis, the epidermis is not vascularized. It mainly consists of keratinocytes, with an interdigitating network of Langerhans cells (LCs) and melanocytes [25]. The epidermis comprises of proliferating keratinocytes at the basal lamina that differentiate towards the skin surface. Keratinocytes express nectin-4 and expression levels increase during differentiation. It is known that keratinocytes are susceptible to MV infection [26]. The top layer of the epidermis, the stratum corneum, consists of a layer of dead keratinocytes called corneocytes. Interestingly, immune cells and nutrients can only reach the epidermis by migration and diffusion, respectively, from the superficial dermis through the basal lamina. The pilosebaceous unit begins at the epidermis and extends into the dermis, where the surrounding tissue is usually better vascularized. Therefore, tissue-resident lymphocytes are often seen in close association with these structures [23]. Hair follicles are mainly constituted of keratinocytes that express high levels of nectin-4 explaining their propensity for MV infection [27].

During viremia, systemic dissemination of MV is mediated by circulating MV-infected CD150^+^ lymphocytes. However, how these cells infiltrate the skin, ultimately resulting in skin rash, remains largely unknown. In this study, we aimed to combine existing and novel information on MV replication in different target cells in the skin to produce one coherent model for the pathogenesis of measles skin rash. We demonstrate that MV infection of lymphoid and myeloid cells in the superficial dermis precedes dissemination to epidermal leukocytes and keratinocytes, which is followed by onset of the typical skin rash.

## Results

### MV skin infection precedes onset of rash in experimentally infected NHPs

**Fig 1:**
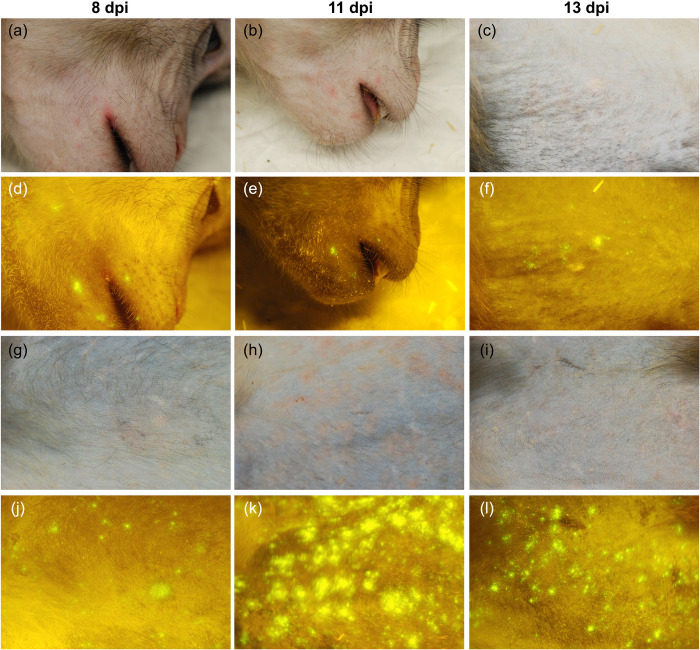
The appearance of MV-infected cells in the skin precedes the appearance of rash. Macroscopic evaluation of MV infection in two cynomolgus macaques: animal #38 (a–f) and animal #37 ((g–l), table S1 in [28]). (a–c; g–i) Normal light: Rash was prominent at 11 dpi. (d–f; j–l) Fluorescence: MV-infected sites (fluorescence) in the skin preceded the rash at 8 dpi and diminished around 13 dpi. Dpi: days post-inoculation.

We retrospectively analyzed data from cynomolgus macaques (*Macaca fascicularis*) inoculated with recombinant MV (rMV) strains expressing enhanced green fluorescent protein (EGFP) [28]. Fluorescent spots, indicating the presence of MV-infected cells, became detectable in the skin around 8 days post-inoculation (dpi), although skin rash only became prominent between 11 and 13 dpi (Fig 1) [29]. We previously reported that by 9 dpi, *i*.*e*. before the onset of rash, lymphocytes and DCs were the predominant infected cell types in the skin [9].

### Phenotypic analysis of MV-infected cells in NHP skin tissues

**Fig 2:**
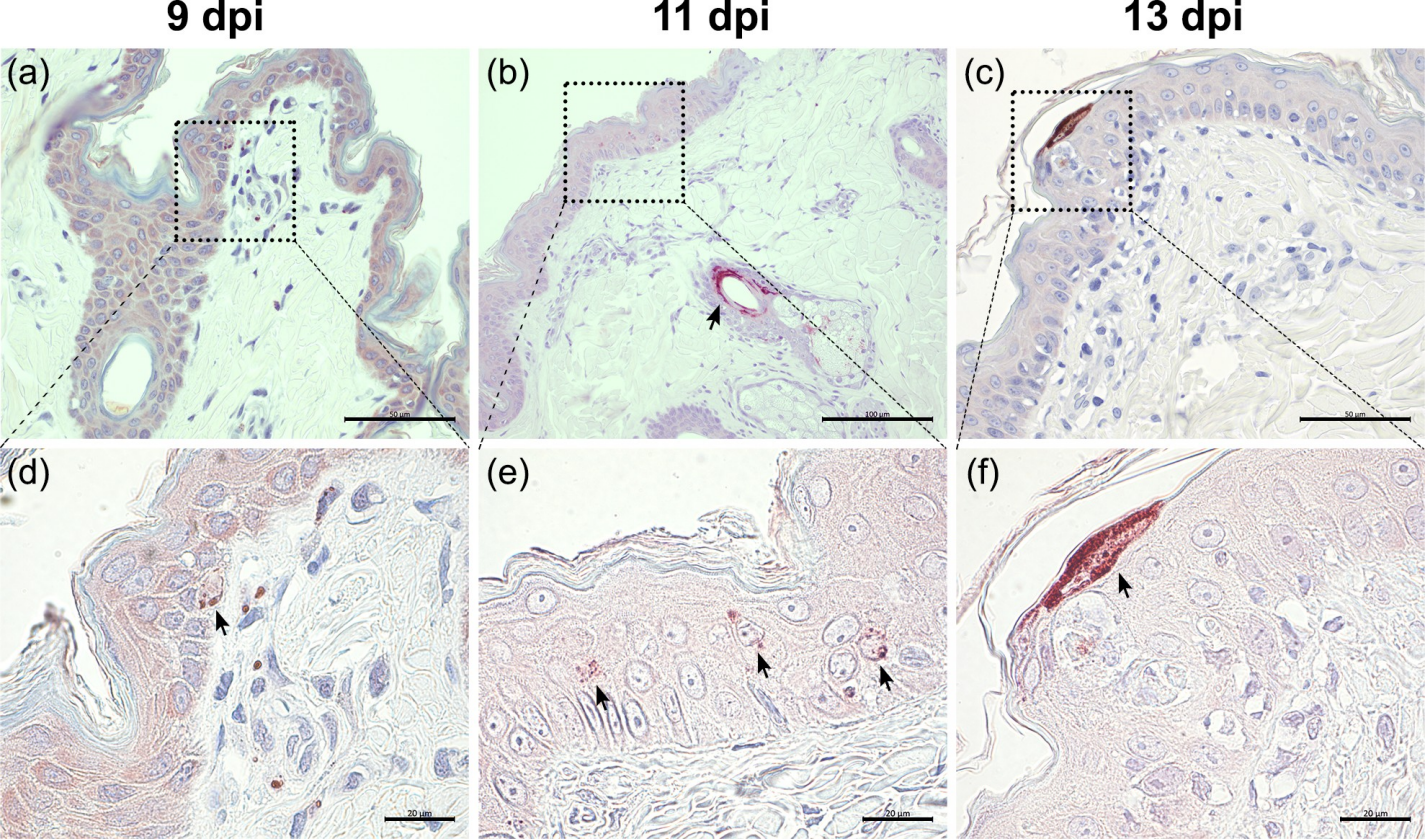
Infection of NHP skin starts in the dermis and spreads to the epidermis. Representative images of immunohistochemical staining of MV-infected macaque skin biopsies collected at 9 (a and d), 11 (b and e) and 13 (c and f) dpi. (a and d) At 9 dpi, most MV N^+^ cells could be found in the dermal papillae, although a few single infected cells were detected in the basal layer of the epidermis. (b and e) At 11 dpi, prominent infection was observed near hair follicles and sebaceous glands (arrow). The infection in the epidermis had progressed further in the suprabasal layers (arrows). (c and f) The infection in the dermis was no longer detected at 13 dpi. The infection in the epidermis had reached the most superficial layers. Scale bars of (a) and (c): 50 μm; Scale bar of (b): 100 μm; Scale bars of (d–f): 20 μm. Dpi: days post-inoculation.

EGFP^+^ skin tissues were collected from the experimentally infected NHPs sacrificed at 9, 11 and 13 dpi. We performed immunohistochemistry on these formalin-fixed and paraffin-embedded skin samples and showed co-localization of EGFP and MV nucleoprotein (N) signals in sequential skin sections, which indicated the presence of MV-infected cells. Representative images of MV-N^+^ cells observed at 9, 11 and 13 dpi are shown in Fig 2A–2F. At 9 dpi, the infected cells were predominantly located in the superficial dermis and in some areas the infection had spread to the epidermis (Fig 2A and 2D). The infection progressed over time and at 11 dpi more MV-N^+^ cells could be found in the dermis and epidermis, most especially around the hair follicles and sebaceous glands (Fig 2B and 2E). By 13 dpi, MV-N^+^ cells were no longer found in the dermis and could only be found in hair follicle or superficial epidermis (Fig 2C and 2F). Edema in the dermis and epidermis could be observed at this time point. Initially, infection in the epidermis was predominantly observed as single infected cells near dermal papillae and later progressed into multiple-cell infection and, rarely, syncytia (S1 Fig) between 9 and 11 dpi.

To assess the location and the phenotype of MV-infected cells in the skin, we performed dual-labeling indirect immunofluorescence (IIF) on these sequential skin sections. CD45^+^ leukocytes were present in the superficial dermis, most especially in or around blood vessels, hair follicles and sebaceous glands and, to a lesser degree, in the epidermis. Some of these CD45^+^ leukocytes were CD3^+^ T cells that were located in the reticular dermis, while some others were S100A8/A9-complex^+^ (MAC387) macrophages that were abundantly present in the superficial dermis, especially in or around the blood vessels, hair follicles or sebaceous glands. CD31^+^ endothelial cells of the blood vessels were exclusively detected in the dermis. In contrast, cytokeratin^+^ cells were exclusively detected in the epidermis and pilosebaceous units.

**Fig 3:**
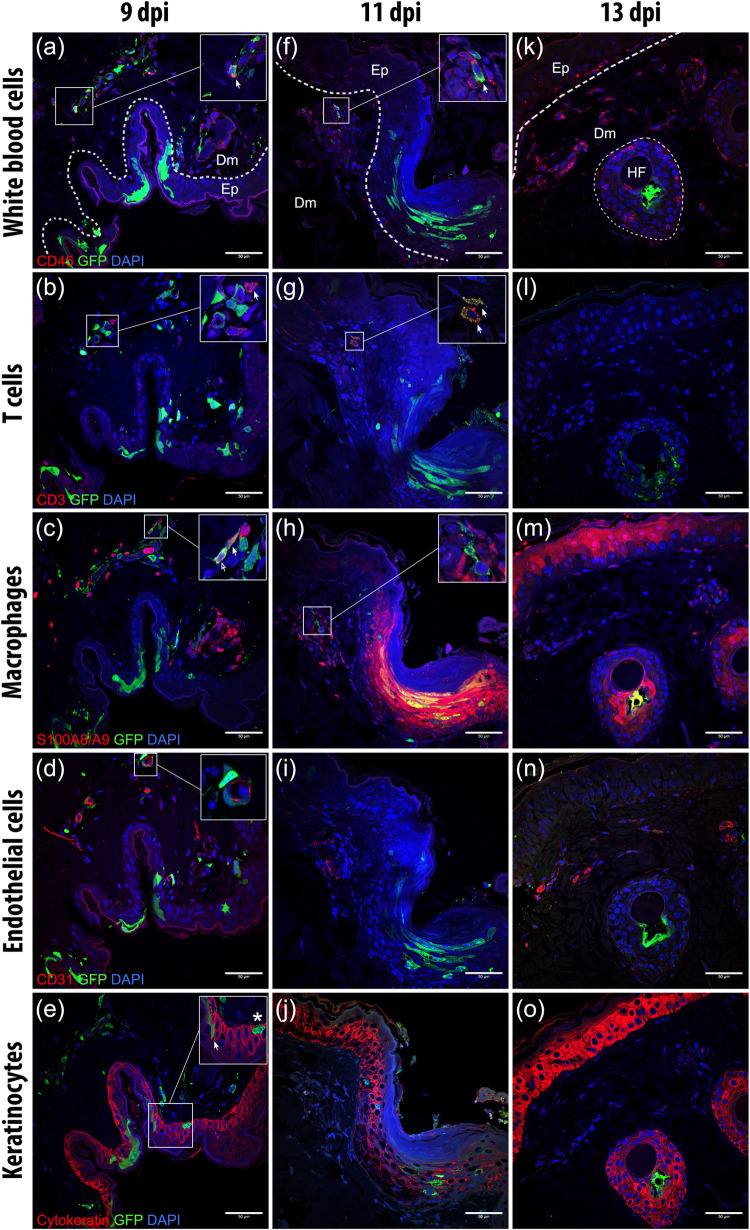
Phenotype of MV-infected cells in the dermis and epidermis throughout the course of infection. Serial sections of skin (top to bottom) of three macaques (left to right) euthanized at three different time points (indicated at the top). The sections were double-stained with antibodies to EGFP (green) and several cell-specific markers (red), as indicated on the left of each row. Dashed lines indicate the basement membrane that separates the dermis (Dm) and the epidermis (Ep). MV-infected cells (green) could be observed in the dermis at 9 dpi. The progression of the infection in the same skin tissue at this time point differed between different sites: either MV-infected cells were found strictly in the dermis or the infection had spread to the epidermis. (a–e) Representative sequential images of MV infection that had progressed to the epidermis at 9 dpi. (a) MV-infected CD45^+^ leukocytes (inset, arrow) could be detected in the superficial dermis. (b) Some of these MV-infected leukocytes were CD3^+^ T cells, which were present in the dermis, mostly in reticular dermis, with speckled GFP signal in their cytoplasm (inset, arrow). (c) MV-infected S100A8/A9 complex^+^ (MAC387) macrophages (inset, arrow) were also found abundantly in the superficial dermis. (d) MV-infected cells in the dermis were often found in or around CD31^+^ blood vessels (inset). (e) In the epidermis, MV-infected cells were mostly keratinocytes (inset, arrow), although MV-infected non-keratinocyte cells (inset, asterisk) were observed in the basal epidermis. (f–j) Representative sequential images of MV infection at 11 dpi. (f) MV-infected leukocytes (inset, arrow), which were (g) T cells in the dermal papillae (inset, arrow), were in close proximity to (h) uninfected macrophages (inset) and (i) blood vessels. (j) The infection in keratinocytes had progressed apically and laterally. MV-infected keratinocytes and the surrounding uninfected keratinocytes expressed S100A8/A9 complex at this time point. (k–o) Representative sequential images of MV infection at 13 dpi. MV-infected cells had mostly disappeared from the dermis at 13 dpi. (k) The dermis and epidermis were filled with leukocytes. (l) No T cells could be observed in the dermal papillae. (m) Macrophages were present abundantly in this area. (n) At this time point, no MV-infected endothelial cells could be observed in the dermis despite their close proximity to (o) some MV-infected keratinocytes in the hair follicle (HF) area. Scale bar: 50 μm. Dpi: days post-inoculation.

MV-EGFP^+^ cells could be found as early as 9 dpi in the dermal papillae. These were predominantly leukocytes (Fig 3A), mostly T cells or macrophages (Fig 3B and 3C). Some MV-infected cells were present in or surrounding blood vessels (Fig 3D). In some areas in which the infection was more progressed the infection had spread to the epidermis, even into the most superficial layers (Fig 3E). The MV-infected leukocytes were still detectable in the dermis on day 11, sometimes in close proximity to uninfected leukocytes, mostly macrophages (Fig 3F–3H). These cells clustered close to the dermal papillae, where many blood vessels could be found (Fig 3I). Meanwhile, the infection in the epidermis had progressed laterally and apically (Fig 3J). We also observed keratinocytes at the site of infection expressing S100A8/A9 complex (Fig 3H). By 13 dpi, the dermis was almost clear of MV-infected cells and was filled with white blood cell infiltrates, mostly macrophages (Fig 3K–3M). No MV-infected endothelial cells were observed at this time point (Fig 3N). Infection in the epidermis had mostly resolved, although remaining infected follicular keratinocytes could still be detected (Fig 3O). Split and merged multicolor fluorescent images of the insets in Fig 3A–3E are available in S2 Fig.

### Dynamics of MV infection in NHP skin tissues

To assess the dynamics of MV infection and the subsequent clearance in the skin, we counted the number of EGFP^+^ cells in five focal infection sites in the NHP skin tissues (n = 2 animals per time point) at different time points after infection (S3 Fig). The number of MV-infected dermal cells decreased at 11 and 13 dpi. In contrast, the number of infected epidermal cells peaked at 11 dpi before decreasing at 13 dpi. We also counted the number of CD45^+^ leukocytes in the dermis and the epidermis of these five focal infection sites, and found that these increased from 9 to 13 dpi. In the epidermis, the number of CD45^+^ cells increased at 11 and 13 dpi, indicating infiltration into this tissue to clear the infected cells during this period. However, we observed high variation in numbers of infected cells in and around foci of infection, both within and between animals.

**Fig 4:**
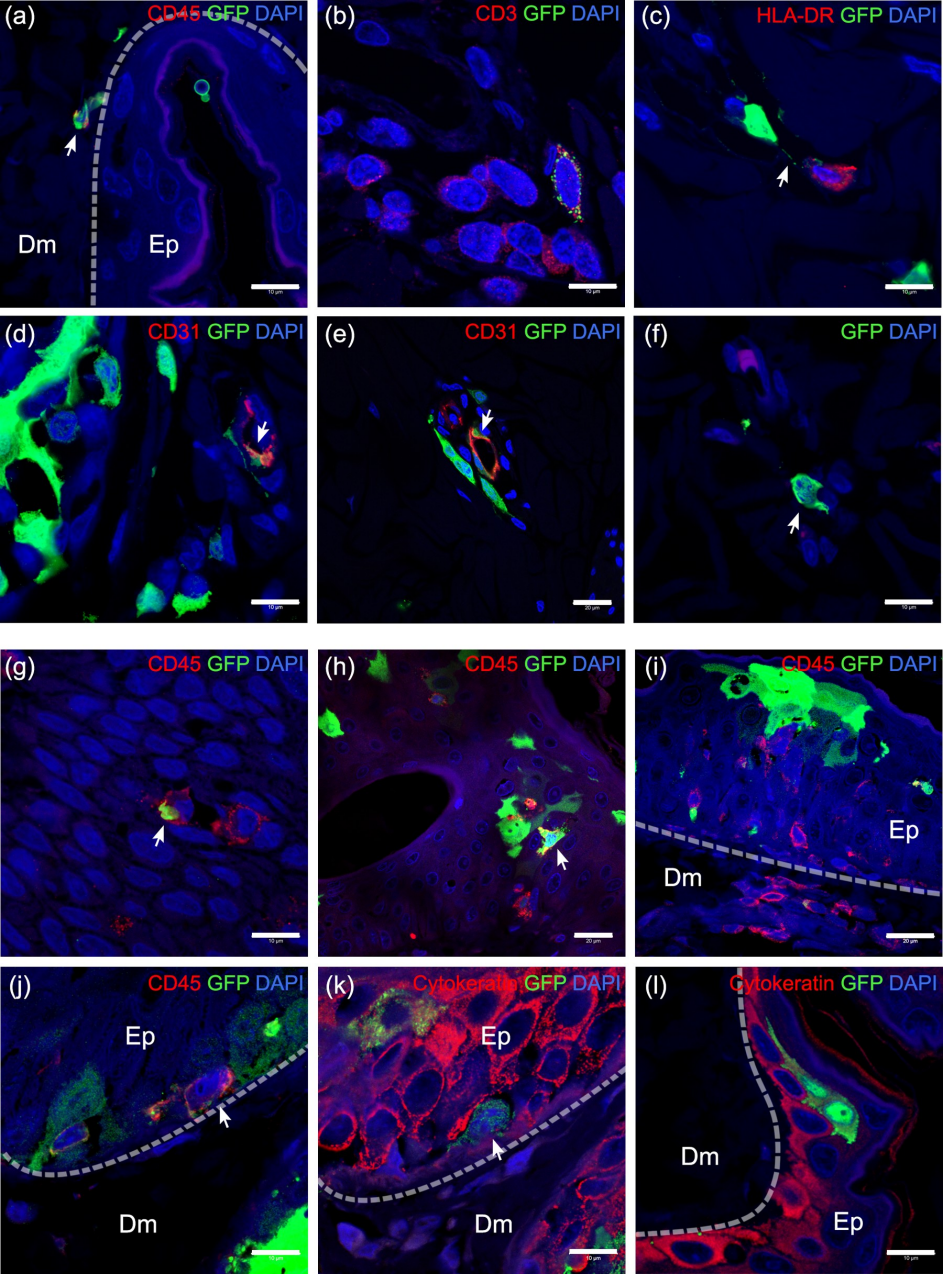
Location of MV-infected cells and interaction with proximal cells. (a–f) MV-infected cells in the dermis and (g–l) in the epidermis. Dashed line indicates the basement membrane that separates the dermis (Dm) and the epidermis (Ep). (a) MV-infected CD45^+^ leukocytes (arrow) in the dermis, especially near the basal layer of the epidermis. (b) MV-infected CD3^+^ T cells (speckled green), although mostly found in the dermal papillae at 9 and 11 dpi, were found in reticular dermis at 13 dpi, surrounded by uninfected T cells. (c) MV-infected cell in the dermis interacted with HLA-DR^+^ APC, forming a long EGFP^+^ dendrite (arrow). (d) More often, MV-infected cells (arrow) located around or in blood vessels and, (e) rarely, MV-infected endothelial cells (arrow) could be found together with those cells. (f) Spindle- or dendritic-like MV-infected cells were negative for all tested cell markers. (g–i) In the epidermis, MV-infected leukocytes could be found since 11 dpi, either interacting with (g) other leukocytes or (h) other MV-infected epidermal cells. (i) Leukocytes appeared to infiltrate the MV-infected cells in the epidermis. (j–k) Serial slides of MV-infected epidermis at 13 dpi. (j) MV-infected leukocytes that were (k) negative for cytokeratin marker could be found in the basal layer of the epidermis. These cells were in close proximity to infected keratinocytes (k). (l) MV-infected keratinocytes in the absence of other infected cells. Scale bars of (a–d), (f), (g) and (j–l): 10 μm; Scale bars of (e) and (h–i): 20 μm. Dpi: days post-inoculation.

Variation in the progression of infection at different sites suggested that time was not the only factor in the pathogenesis of MV skin rash. The location in which MV-infected cells were found and the density and mobility of susceptible neighboring cells at that site also seemed to play an important role. We observed MV-infected leukocytes in dermal papillae or close to the basal epidermis (Fig 4A). MV-infected T cells could often be found near the dermal papillae around 9 and 11 dpi, but became scarce at 13 dpi and could only be observed in the reticular dermis. At this late time point, the MV-infected T cells were found surrounded by uninfected T cells (Fig 4B). Close interaction could also be observed among MV-infected cells with HLA-DR^+^ antigen-presenting cells (APCs), for example through a long, EGFP^+^ dendrite (Fig 4C). We observed MV-infected cells surrounded by or in close proximity to endothelial cells (Fig 4D) at 9 dpi at the site where the infection had progressed further. Very rarely, in the same site, we found MV-infected CD31^+^ endothelial cells near other infected cells (Fig 4E). We also observed MV-infected cells in the dermis that were negative for markers of leukocytes, APCs, endothelial or epithelial cells and appeared to be spindle- or dendritic-like cells (Fig 4F). Multicolor fluorescent images of dermis of experimentally infected NHPs are available as split and merged images in S4 Fig.

In the epidermis, a number of MV-infected leukocytes were observed at 11 dpi, accompanied by infiltration of uninfected leukocytes to the site of infection (Fig 4G–4I). Many of these were macrophages (S4). Infrequently, some of these MV-infected leukocytes were negative for keratinocyte, macrophage and T cell markers (Fig 4J and 4K). These cells were found in close proximity to keratinocytes, which were also positive for MV infection (Fig 4K), although keratinocyte infection could still be detected despite the absence of MV-infected leukocytes in the observed two-dimensional plane (Fig 4L). Multicolor fluorescent images of epidermis of experimentally infected NHPs are available as split and merged images in S5 Fig.

### *Ex vivo* MV infection of human skin sheets results in higher infection levels in the dermis than in the epidermis

**Fig 5:**
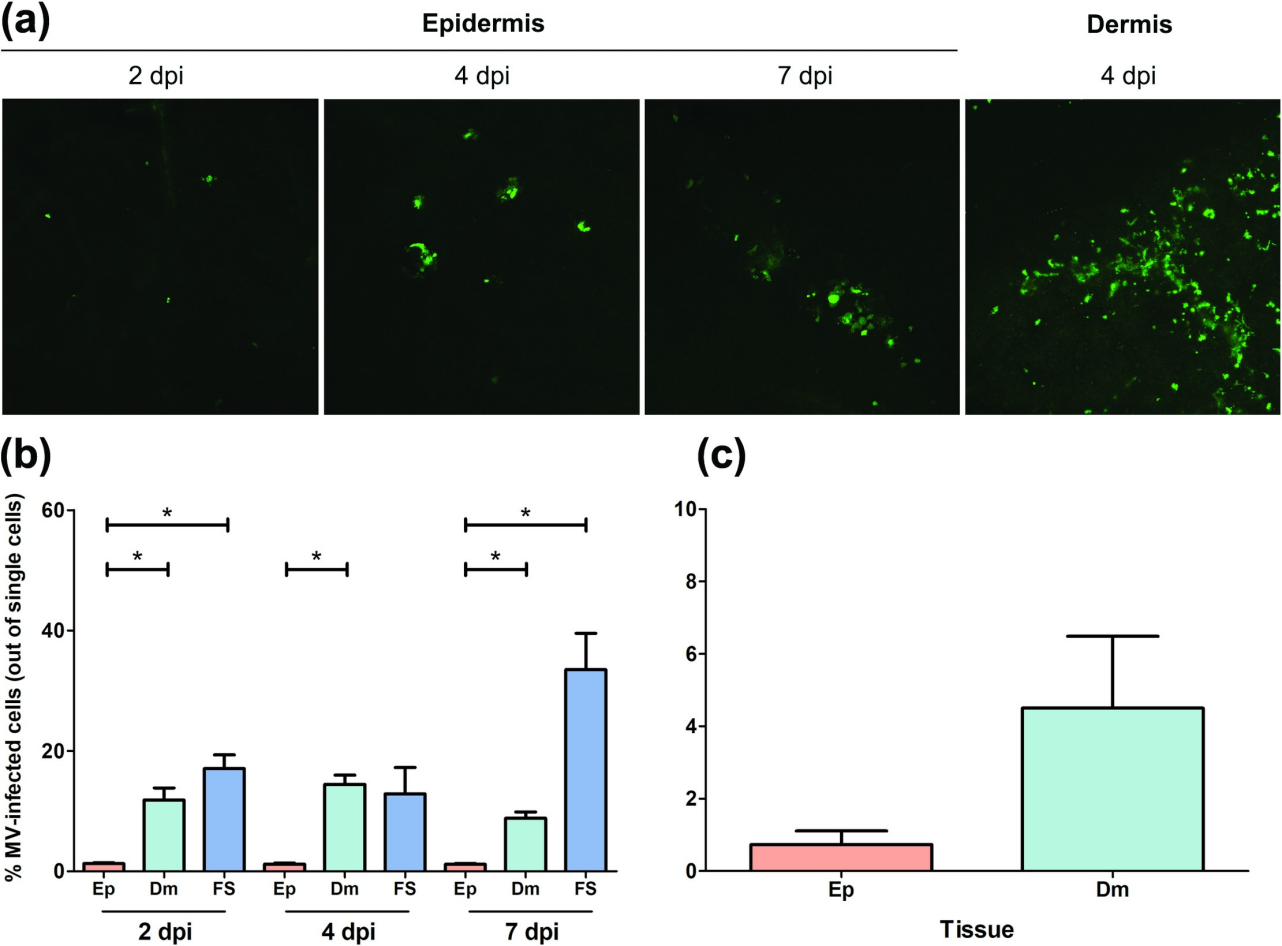
*Ex vivo* MV infection of epidermis, dermis or full skin sheets. (a) Representative images of MV-infected cells in the dermis and the epidermis. MV^+^ cells (green) were detectable as early as 2 dpi in the epidermis and were present in higher numbers in the dermis than in the epidermis. (b) Percentages of emigrant MV-infected cells in supernatants of *ex vivo* cultured epidermis (Ep), dermis (Dm) or full skin (FS) (n = 3 donors) at 2, 4 and 7 dpi, as determined by flow cytometry. (c) *Ex vivo* MV-inoculated full skin sheets were kept in culture up to 4 dpi before enzymatically separated into epidermal and dermal sheets. These sheets were further kept in individual culture up to 7 dpi. The percentages of emigrant MV-infected cells from the supernatants of the separated epidermal and dermal sheets were determined by flow cytometry at 7 dpi. All experiments were performed in triplicate. Dpi: days post-inoculation. *, *P* < 0.05.

Based on the observations in NHPs, we hypothesized that the dermis is the primary target site for MV skin infection. To test this hypothesis, we *ex vivo* inoculated human full skin or enzymatically-separated epidermal and dermal sheets with rMV based on a wild-type MV strain Khartoum-Sudan (KS) expressing the fluorescent reporter protein Venus from an additional transcription unit in position 3 of the viral genome (rMV^KS^Venus(3)) [30]. We observed that Venus^+^ cells could be detected by inverted laser scanning microscopy as early as 2 dpi, with higher infection levels in the dermis than the epidermis (Fig 5A). The percentages of emigrant MV-infected cells in the supernatants of the skin sheet cultures were determined by flow cytometry. In accordance with the observation of Venus^+^ cells by microscopy, the percentages of MV-infected emigrant cells were higher in the dermis and full skin than in the epidermis (Fig 5B).

To confirm this observation, we enzymatically-separated the MV-inoculated full skin into epidermis and dermis at 4 dpi. The separated sheets were subsequently cultured individually up to 7 dpi. The percentage of emigrant MV-infected cells in the supernatants of these separated skin sheet cultures were determined by flow cytometry. We observed a trend towards higher percentages of MV-infected emigrant cells in supernatants of the separated dermis sheets as compared to their epidermal counterparts (Fig 5C).

Previous studies showed that mature LCs are susceptible to MV infection and Langerin can act as an attachment receptor, but not entry receptor, for the virus [21]. To determine whether LCs play a role in MV epidermal infection as initial target cells, we performed dual-labeling IIF on human epidermal sheets from healthy donors infected with rMV^KS^Venus(3). We found that despite the abundant presence of LCs in the epidermal sheets, none of these were Venus-positive (S6 Fig).

### Human primary keratinocytes are susceptible to *in vitro* MV infection in a nectin-4-dependent manner

**Fig 6:**
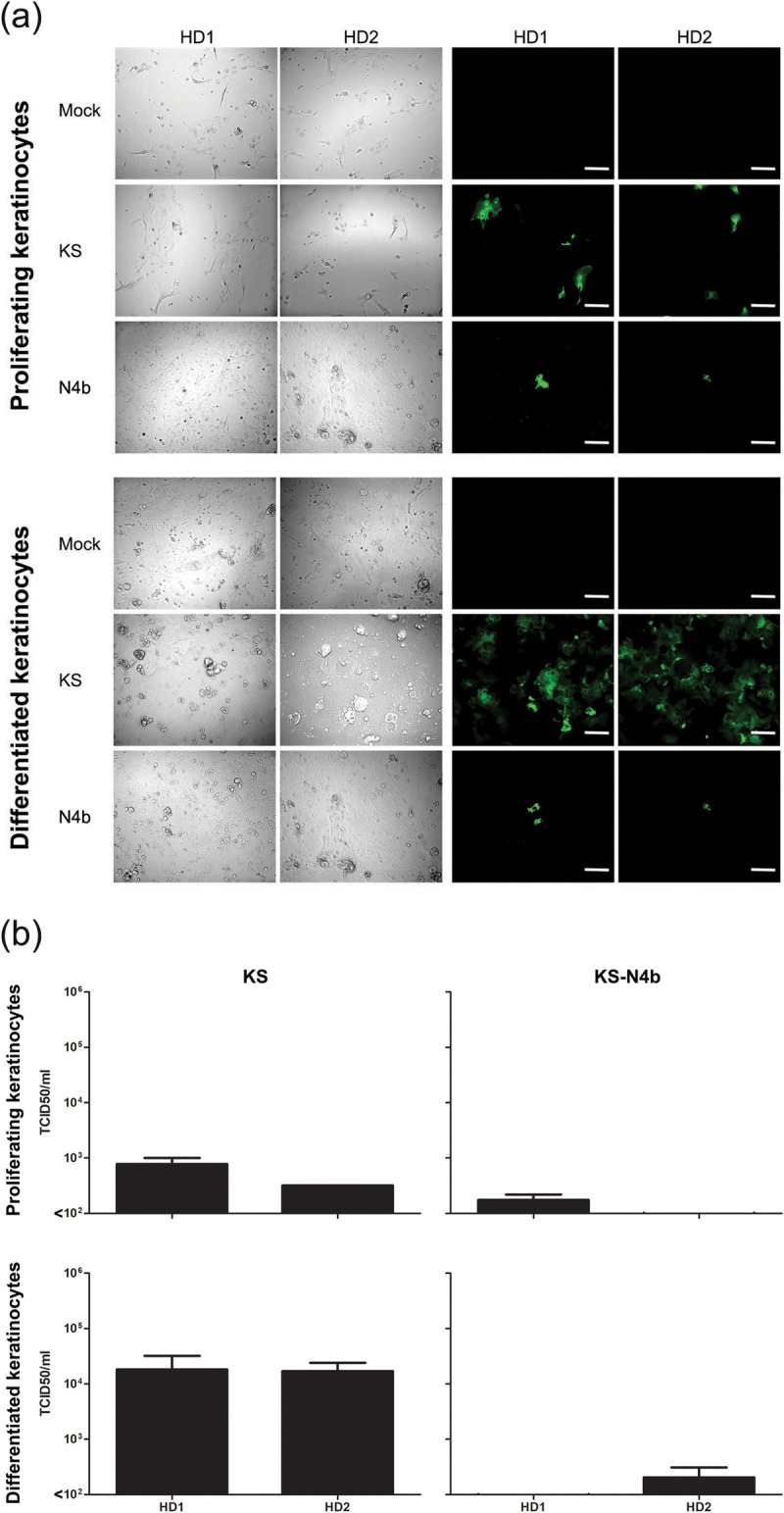
Susceptibility and permissiveness of proliferating and differentiated human primary keratinocytes to *in vitro* MV infection. (a) Higher numbers of infected keratinocytes (green) were detected in differentiated than in proliferating keratinocyte cultures, regardless of MV strain. NCI-H358: human broncho-alveolar carcinoma cell line; BLCL: EBV-transformed B-lymphoblastoid cell line. Scale bars: 200 μm. (b) Differentiated keratinocytes produced higher number of infectious cell-free virus than proliferating keratinocytes. All experiments were done in duplicate. HD1 or HD2: primary keratinocyte culture from healthy donor 1 or 2; KS: rMV^KS^Venus(3); KS-N4b: rMV^KS-N4b^EGFP(3).

We investigated the susceptibility and the permissiveness of human primary keratinocytes from two healthy donors to *in vitro* MV infection. In agreement with previously published data, nectin-4 expression on the cell surface was highest in differentiated keratinocytes, as demonstrated by flow cytometry (S7 Fig) [27]. To determine whether the proliferating and differentiated keratinocytes were susceptible to MV infection, we inoculated them with rMV^KS^Venus(3) or a strain engineered to be unable to recognize nectin-4 (the ‘nectin-4-blind (N4b)’ strain rMV^KS-N4b^EGFP(3)) [11] at a multiplicity of infection (MOI) of 1. After 48 hours, we observed higher frequencies of fluorescent cells in differentiated than in the proliferating cells (Fig 6A). Infection of keratinocytes with the nectin-4-blind MV resulted in low numbers of single infected cells.

To assess whether the infected keratinocytes also produced cell-free virus and were thus capable of spreading the infection, the supernatant of the MV-infected keratinocytes was collected and the titer of cell-free virus in the supernatant was assessed [31]. Cell-free MV was detectable in the culture supernatant of the infected proliferating and differentiated keratinocytes. Virus titers in supernatants of differentiated keratinocytes were higher than those in supernatant of proliferating keratinocytes (Fig 6B).

## Discussion

**Fig 7:**
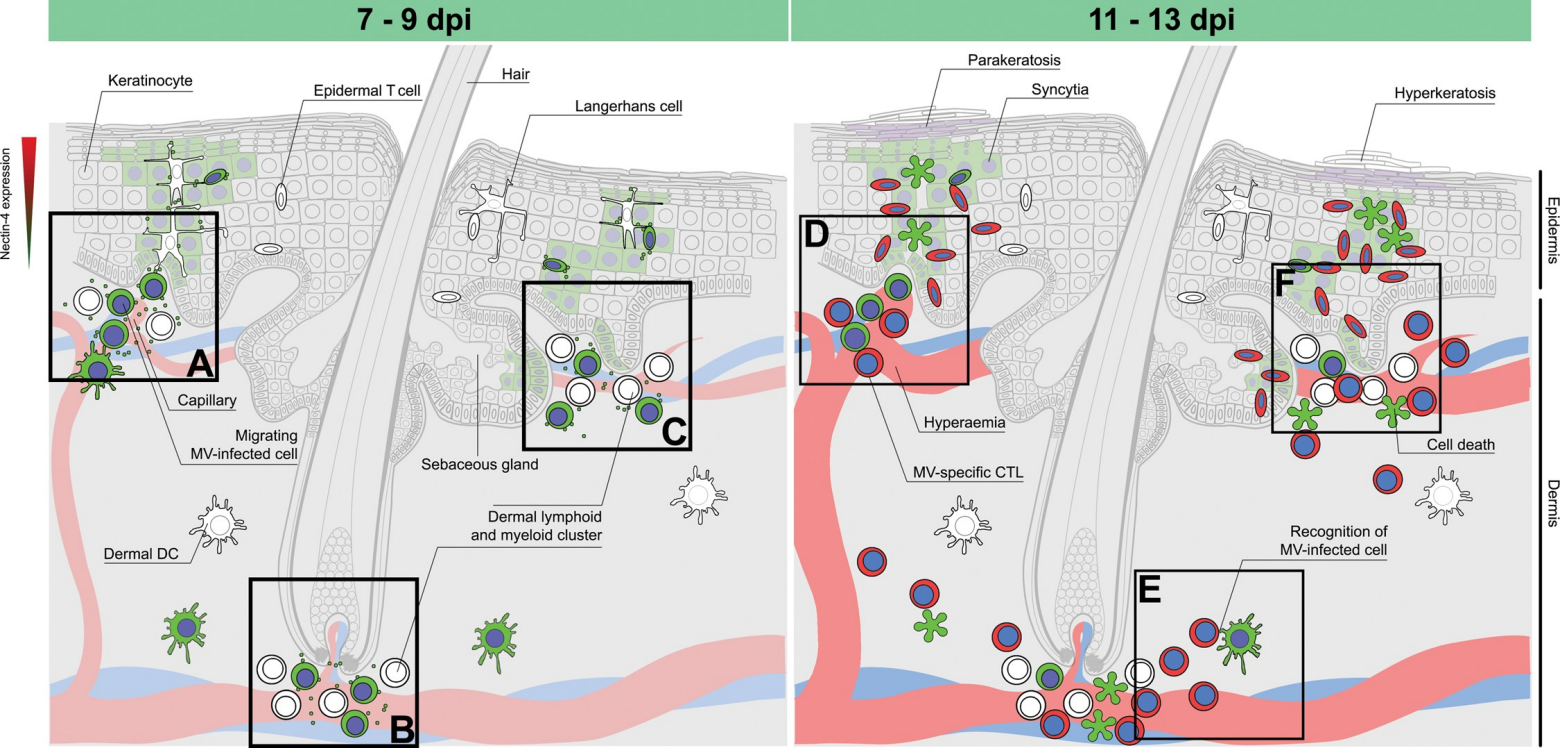
Model for the pathogenesis of measles skin rash. During viremia, MV-infected T cells and macrophages migrate to the dermis via the capillaries and interact with (a) tissue-resident lymphoid and myeloid cells and epidermal LCs residing near the basal lamina. This interaction leads to the infection of surrounding CD150^+^ tissue-resident immune cells and nectin-4^+^ epithelial cells. Alternatively, MV-infected T cells and macrophages migrate in close proximity to: (b) the hair follicle or (c) the sebaceous gland via the capillary, where they infect an aggregate of tissue-resident T cells and macrophages, and further spread the infection to nearby keratinocytes and LCs. Infection of basal keratinocytes leads to lateral and apical spread of the virus to the superficial layers of the epidermis. Several days later, (d) hyperemic responses allow the recruitment of MV-specific CD8^+^ cytotoxic T cells and macrophages, resulting in (e) recognition and (f) clearance of the infected cells. Hyperemia and subsequent edema are the histological correlates of maculopapular erythematous measles rash. The time range given at the top of the figure is based on observations from experimentally infected NHPs. Dpi: Days post-infection.

The pathogenesis of MV skin rash is not well understood. Here, we aimed to identify the cell types involved in MV infection of skin, and the kinetics of viral dissemination in relation to onset of rash. Based on our findings, combined with previously published observations, we postulate a model that describes the progression of MV skin infection and the development of measles rash (Fig 7). The model takes viral tropism, location, interaction and motility of the susceptible cells, as well as the virus-specific immune responses into account. MV-infected cells enter the superficial dermis through the blood vessels and spread the infection to the tissue-resident dermal T cells, APCs and spindle- or dendritic-like cells around 7 days after infection. The infection progresses several days later to the adjacent epidermal areas, where the virus is transmitted to the basal keratinocytes. As basal keratinocytes differentiate apically to the suprabasal layers and their nectin-4 expression increases, the virus spreads apically and laterally and the infected keratinocytes subsequently form syncytia. Infection of dermal endothelial cells was very rare, but not completely absent. We speculate that the infection is subsequently cleared around 13 days after infection by infiltrating immune cells, which first migrate into the dermis and later into the epidermis.

The dermis contains several potential target cells for MV infection. Due to vascularization, the dermis is filled with CD150^+^ lymphoid and myeloid cells that traffic through or reside in the tissue. CD4^+^ and CD8^+^ T cells localize and move differently in the skin [32]. Slow-moving CD8^+^ resident memory T cells (T_RM_) reside in the epidermis and hair follicles, while highly motile CD4^+^ effector memory T cells (T_EM_) migrate into the dermis and recirculate systemically [33]. We detected MV-infected T cells in the dermis from 9 dpi onward, but never in the epidermis at that time point. Previous studies have shown that CD4^+^ T_EM_ cells are highly susceptible to MV infection [28, 34]. Interaction of MV-infected T cells with skin-resident APCs may result in further cutaneous spread. T cells have been described in human skin to cluster with APCs around appendages, such as hair follicles [35–37]. We did not observe such T cell clusters in NHPs, most likely due to T cell depletion that occurs systemically and peaks around 9 dpi [28]. Whether this depletion leads to the loss of pre-existing skin-resident memory T cells remains to be studied. Additionally, we and others have observed MV-infected T cells and APCs around hair follicles and sebaceous glands [15, 38], which are surrounded by nectin-4^+^ epithelial cells [27]. The close proximity of these infected cells to the basal keratinocytes may lead to the spread of MV infection from the dermis to the epidermis.

The epidermis consists predominantly of keratinocytes, which express nectin-4 and are susceptible to MV infection [26]. We were not able to demonstrate the expression of nectin-4 in the NHP epidermis due to the lack of cross-reactive antibodies. However, in accordance with a previous study, we show primary human keratinocytes express nectin-4 and its expression is upregulated upon differentiation [27]. We show here that nectin-4 expression plays a role in the susceptibility of keratinocytes to *in vitro* MV infection: higher expression of nectin-4 resulted in higher susceptibility. We also inoculated primary keratinocytes from a patient affected by ectodermal dysplasia-syndactyly syndrome (EDSS1, OMIM 613573), an autosomal recessive disorder caused by mutations in the nectin-4 encoding gene *PVRL4* [27]. We observed that despite the strongly reduced expression of nectin-4 in this patient, the keratinocytes were still susceptible and permissive to *in vitro* MV infection, albeit at lower levels compared to the healthy donors (S8 Fig).

Due to the focal nature of MV skin infection, the progression of MV infection in the epidermis varied in different sites in experimentally infected NHPs. At 9 dpi, epidermal infection was predominantly observed as single infected cells in the basal epidermis. However, in some sites where the progression of the infection had developed further in the suprabasal epidermis into multiple-cell infection and syncytia. Interestingly, we also observed expression of S100A8/A9 complex (MAC387) from 11 dpi and 13 dpi in the epidermis of experimentally infected NHPs. Although the expression of S100A8/A9 complex is often restricted to myeloid cells, its induction has also been described in hyperproliferative and differentiated keratinocytes, such as during wound healing or in psoriasis lesions [39, 40]. Although MV infection in the epidermis could be observed as early as 9 dpi, the expression of S100A8/A9 complex by keratinocytes was only observable at 11 and 13 dpi. Moreover, this complex was expressed not only by MV-infected cells, but also the surrounding keratinocytes. In uninfected areas at 11 and 13 dpi, the keratinocytes were negative for S100A8/A9 complex. Altogether, these observations suggest that the expression of S100A8/A9 complex was induced by focal MV infection in the epidermis. Focal hyperkeratosis and parakeratosis have been reported in measles skin biopsies [15]. Whether the expression of this complex leads to hyperproliferation and differentiation of keratinocytes in an MV-infected site, and subsequently leads to measles-associated hyperkeratosis and parakeratosis remains to be determined.

Beside the keratinocytes, another cell type of interest in the epidermis is the LC, a subset of DCs. Although we could not observe MV-infected LCs in our human skin *ex vivo* model, LCs are known to be susceptible to MV infection [21, 41, 42]. The activation status of the cells also determines their susceptibility, since immature LCs are not susceptible to MV infection, while mature ones are [21]. This offers an explanation to why the LCs were not susceptible to MV infection in our *ex vivo* model: the cells might still have been in their immature state. We were not able to identify LCs in cynomolgus macaque skin tissues due to the unavailability of cross-reactive antibodies. The susceptibility of LCs to MV infection *in vivo* and their role in the pathogenesis of measles skin rash remain to be determined. Additionally, LCs express Langerin that can act as an attachment, but not entry, receptor to MV [21] and thus can indirectly introduce MV infection to the epidermal keratinocytes by acting as an attachment hub for the virus from the dermis. Although we were able to clearly identify APCs and T cells in the dermis, we were not able to detect HLA-DR^+^ or CD3^+^ cells in the epidermis.

DCs and macrophages occupy the dermis as professional APCs and phagocytes, respectively. Macrophages are present in high numbers and are associated with blood or lymphatic vessels, while dermal DCs have been found to form clusters with T cells, suggesting the presence of an inducible structure of macrophages, DCs and T cells that may function as a skin-associated lymphoid tissue [43, 44]. In the respiratory tract, DCs and macrophages act as Trojan horses during MV infection by spreading the virus to the lymphocytes in draining lymph nodes [8, 20, 45–47]. Migrating or patrolling MV-infected DCs and macrophages may play the same role in the skin as they do in the respiratory tract. However, these cells may also play a crucial role as innate immune cells that inhibit infection. Close communication of MV-infected DCs and macrophages with T cells can lead to activation of MV-specific immune responses and subsequently to the development of rash. The role of these immune responses in the development of rash has been highlighted in immunocompromised patients with MV infection that do not develop skin rash [14].

Blood vessels and capillaries run through the dermis. The capillaries penetrate into the dermal papillae, from where the distance to the epidermis is minimal, and the distribution of the capillary loops differs according to the type of the skin. The capillary bed consists of an arteriole, which gives rise to metarterioles and subsequently hundreds of capillaries. The capillaries provide the dermis and epidermis with nutrition and oxygen, and connect to venous capillarioles and further to a venule. Inflammation due to infection may cause prolonged vasodilatation and increased capillary permeability. This hyperemic reaction allows the release of chemokines by skin-resident cells, such as memory immune cells and keratinocytes, that leads to the infiltration of various immune cells, such as macrophages and lymphocytes. Alternatively, skin-resident cells can induce the release of chemokines that leads to hyperemia. The vasodilatation results in swelling, but not leakage, of tissue capillaries with oxygenated blood and gives the appearance of superficial reddening of the skin (erythema) and edema [48]. Given that measles rash is described as maculopapular (*i*.*e*. small with raised bumps) and erythematous (*i*.*e*. red), and edema can be observed in MV-infected skin [15], we speculate that hyperemia is responsible for the appearance of the erythematous maculopapular rash. Although theoretically it is possible to investigate the presence of hyperemia in our *in vivo* model by showing an increased number of erythrocytes in the cutaneous blood vessels, we could not perform the calculation fairly, since the animals were sacrificed by exsanguination.

MV infection in the skin gives a unique appearance of rash compared to other viral exanthemata. Rubella rash, for example, has been described as macroscopically similar to measles rash, since it gives a pink-reddish “rubelliform” maculopapular rash. However, in rubella, viral infection takes place deeper in the dermis, in contrast to measles skin infection that occurs more superficially in the dermis and the epidermis. Infection of the keratinocytes, which is typical for measles rash, does not occur during rubella virus infection [49]. In contrast, varicella zoster virus (VZV), as a representative of the *Herpesviridae* family member, has similar target cells in the dermis and epidermis as MV, but displays a different type of rash. VZV infects perivascular macrophages and DCs as well as keratinocytes, but the infection leads to the appearance of spots that turn into itchy blisters [50]. Arboviral exanthemata, on the other hand, have a different route of infection, but often present overlapping outcomes in the skin. Dengue virus is introduced into the body through a mosquito bite and injected into the bloodstream, with spillover to the epidermis and the dermis. This spillover causes infection of LCs and keratinocytes. Dengue virus spreads systemically through the infection of monocytes and macrophages. The virus also causes vascular leakage through infection of endothelial cells, leading to the appearance of minor hemorrhagic lesions [51]. Although petechial rash is one of the clinical manifestations of dengue virus infection, morbilliform rash is also often described during classical dengue fever [52]. Altogether, these findings, including ours, strongly suggest that the appearance of skin rash is closely linked to the viral tropism, the availability and location of susceptible target cells and the subsequent immune responses to clear the infection.

In conclusion, our study offers a comprehensive model for the pathogenesis of measles skin rash: MV-infected lymphocytes and myeloid cells enter the dermis, where the infection spreads to the susceptible cells in the vicinity of dermal papillae, hair follicles, sebaceous glands and blood vessels in the superficial dermis. The infection spreads laterally and apically to the epidermis in a nectin-4-dependent manner. The infection is cleared several days later by infiltrating immune cells, accompanied by the appearance of edema and hyperemia that give the appearance of an erythematous morbilliform rash.

## Materials and methods

### Ethics statement

All NHP samples were derived from previously published studies, and no new experimental infections were performed [28]. Studies involving the use of primary keratinocytes were approved by the local ethics committee, and written informed consent was obtained from the EDSS1 patient and the healthy volunteers [27]. Studies using human skin tissue were performed in accordance with the Amsterdam University Medical Centres (AUMC) institutional guidelines with approval of the Medical Ethics Review Committee of the AUMC, location Academic Medical Centre, Amsterdam, the Netherlands, reference number: W15_089 # 15.0103. All samples were handled anonymously.

### Cells

Culture of normal and EDSS1 primary human keratinocytes was carried out as previously described [27]. Proliferating keratinocytes were cultured till sub-confluence in serum-free keratinocyte growth medium (KGM, Invitrogen) containing 0.15 mM Ca^2+^, and then induced to differentiate by culturing for further 3 days in complete keratinocyte culture medium composed of a 3:1 mixture of Dulbecco’s modified Eagle medium (DMEM) and Ham's F12 media (Invitrogen) containing 10% of foetal bovine serum (FBS), insulin (5 μg/ml), transferrin (5 μg/ml), adenine (0.18 mM), hydrocortisone (0.4 μg/ml), cholera toxin (0.1 nM), triiodothyronine (2 nM), epidermal growth factor (EGF; 10 ng/ml), glutamine (4 mM), and penicillin-streptomycin (50 IU/ml). Epstein-Barr virus- (EBV-) transformed B-lymphoblastoid cell line (BLCL) and human broncho-alveolar carcinoma (NCI-H358) cell lines were grown in RPMI-1640 medium supplemented with 10% of FBS, 100 IU of penicillin/ml, 100 μg of streptomycin/ml and 2 mM glutamine (R10F medium). Vero cells expressing human CD150 (Vero-CD150) were grown in DMEM supplemented with 10% of FBS, 100 IU of penicillin/ml, 100 μg of streptomycin/ml and 2 mM glutamine (D10F medium) [53]. All cells were cultured in a humidified incubator at 37°C with 5% of CO_2_.

### *Ex vivo* culture of human skin tissues

Residual skin materials were obtained from three adult human donors undergoing correctional surgery and stored at 4°C overnight. The skin was shaved using a dermatome (0.3 mm, Zimmer Biomet). For the preparation of full skin sheets, which consist of dermis and epidermis, the shaved skin was cut into circular sheets (diameter approximately 1 cm) using a skin biopsy punch and cultured in IMDM supplemented with 10% of FBS, 100 IU of penicillin/ml, 100 μg of streptomycin/ml (Invitrogen), 2 mM glutamine and 20 μg/ml gentamicine (Centrafarm) (I10F medium), with the epidermis facing upward. The full skin pieces were stored in a 24-well plate in I10F medium. For the preparation of epidermal sheets, shaved skin was incubated in I10F medium in the presence of 1 U/ml of dispase (Roche Diagnostics) for 1 h at 37°C. The epidermis was separated from dermis using a pair of forceps and cut into circular sheets using a skin biopsy punch. The epidermal, dermal or full skin sheets were cultured in a 24-well plate in I10F medium, with the keratin layer of the epidermis facing upward. Experiments were performed in triplicate.

### Viruses

All recombinant MV strains used in this study were described previously: recombinant MV strain Khartoum-Sudan (KS) expressing the fluorescent protein Venus from an additional transcription unit in position 1 or 3 (rMV^KS^Venus(1) or (3)) [30] was based on wild type viruses. An rMV^KS^ expressing EGFP in position 3 of the viral genome engineered to be unable to recognize nectin-4 (referred to as the ‘nectin-4-blind (N4b)’ rMV^KS-N4b^EGFP(3)) was also included in this study [11]. Virus titers were determined by endpoint titration on Vero-CD150 cells, and were expressed as 50% tissue culture infectious dose (TCID_50_) per ml calculated as described by Reed and Muench [31].

### *In vitro* MV infection

Adherent primary keratinocytes were either inoculated directly or were treated with trypsin-EDTA (0.05%) and inoculated in suspension with the two different rMV strains at an MOI of 1. After 2 h, the suspension cells were washed to remove unbound virus and seeded onto 24-well plates in complete keratinocyte culture medium. After 48 h of infection, the cells were observed under an inverted-laser scanning LSM-700 microscope (Zeiss) and the infection percentages were assessed by flow cytometry.

### Ex vivo MV infection

Full skin pieces, dermal or epidermal sheets were inoculated with cell-free rMV^KS^Venus(3). Briefly, 200 μl of pure virus stock (3.7 × 10^6^ TCID_50_/ml) was added to each well of a 24-well plate, and the skin sheets were added on top of the liquid with the epidermis facing upwards. While full skin and epidermal sheets remained afloat, dermal sheets tended to sink and both apical and basolateral surfaces were exposed to virus. After 2 h at 37°C, I10F medium was added to the wells. The progression of infection was observed at 2, 4 and 7 dpi under the inverted laser scanning microscope. At 4 dpi, mock- and MV-inoculated full skin sheets were incubated in I10F medium in the presence of 1 U/ml of dispase (Roche Diagnostics) for 1 h at 37°C and separated into epidermal and dermal sheets. The separated sheets were cultured individually in a 24-well plate in I10F medium up to 7 dpi.

### Measurement of MV production by infected keratinocytes

Supernatant of MV-infected keratinocytes was titrated into 96-well plates containing Vero-CD150 cells (1 × 10^4^ cells/well). The titer of the virus was expressed as TCID_50_/ml and calculated as described above.

### Flow cytometry

Flow cytometry was performed using a BD FACSCanto II, unless mentioned otherwise. Primary keratinocytes were labelled with nectin-4^PE^ antibody (clone 337516; R&D Systems) to assess the expression of nectin-4. Isotype control (Isotype^PE^, clone 27–35, BD Biosciences) antibody was included to assess the level of background staining. NCI-H358 cells and BLCL were included as positive and negative controls for nectin-4 expression, respectively. All cells were fixed with 2% of PFA prior to measurement of the percentage of cells expressing the virus-encoded fluorescent protein. Mock-infected cells were included as infection control. Supernatants from full skin pieces, dermal or epidermal sheets (n = 3 donors) were isolated at 2, 4 and 7 dpi and emigrant cells were isolated after undergoing centrifugation. Mock-infected tissues were included as infection control. Percentages of MV-infected emigrant cells were measured by flow cytometry using a BD FACSLyric. Gating strategy used in the flow cytometry analyses is shown in S9. Data was acquired with BD FACSDiva or FACSSuite software and analyzed with FlowJo software.

### *In situ* analyses

Immunohistochemistry was performed on one to three formalin-fixed, paraffin-embedded skin tissues originating from different EGFP^+^ regions (abdomen, eyelid or lip) from experimentally infected NHPs (n = 2 animals per time point) euthanized at 9, 11 and 13 dpi using monoclonal antibodies directed to MV N protein (clone 83KKII, Chemicon [54]) or rabbit polyclonal antibody directed to GFP (Invitrogen). Goat anti-mouse IgG1 or goat anti-rabbit antibody conjugated with biotin was included as secondary antibody. Streptavidin-horseradish peroxidase was added for signal detection. Dual-labeling IIF assays on sequential skin slides of experimentally infected NHPs were performed using mouse monoclonal antibodies directed to CD45 (clone 2B11+PD7/26; DAKO), CD3 (clone F7.2.38; DAKO), CD31 (clone JC70A; DAKO), cytokeratin (clone AE1/AE3; DAKO), S100A8/A9 complex (clone MAC387; Abcam), or HLA-DR (clone L243; BioLegend) in combination with rabbit polyclonal antibody directed to GFP. Goat anti-rabbit-IgG-Alexa Fluor (AF)488 (Invitrogen) and goat anti-mouse IgG-AF594 (Invitrogen) were included as secondary antibodies. Formalin-fixed, paraffin-embedded tissues were sectioned at 3 μm, deparaffinized and rehydrated prior to antigen retrieval. Antigen retrieval for MV N protein staining was performed in the presence of 0.1% protease in pre-warmed PBS for 10 minutes at 37°C. Antigen retrieval for other stainings was performed in citrate buffer (10 mM, pH = 6.0) with heat induction. Sections were incubated with primary antibody overnight at 4°C before incubation with secondary and tertiary antibodies. For dual-labeling IIF assays, the slides were mounted with ProLong Diamond Antifade Mountant with DAPI (Thermo Fisher Scientific) prior to fluorescence detection with the inverted laser scanning microscope. Images were obtained using 1–2 times frame averaging and the pinhole adjusted to 1 airy unit. To quantify EGFP^+^ and CD45^+^ cells in NHP skin tissues, five high-power Z-stack fields (400 × magnification) containing MV focal infection sites were arbitrarily selected per animal per time point (n = 2 animals per time point; in total 3 time points). Cell counting to determine the number of EGFP^+^ and CD45^+^ cells in the NHP skin tissues was performed in Fiji software.

### Statistical analysis

Differences between the percentages of infected cells in the human *ex vivo* epidermal, dermal or full skin sheets were analyzed by paired t-test.

## Supporting information

S1 Fig The focal nature of measles skin infection resulted in different progression of infection in the dermis and epidermis. At 9 dpi, MV infection were mostly found in the dermis of experimentally infected NHPs. However, due to the focal nature of MV skin infection, MV-infected cells could sometimes also be detected in the epidermis. The progression of epidermal infection varied in different sites, ranging from only single-cell to multiple-cell infection. A syncytium (ellipse) was observable, albeit rarely, in the epidermis collected at 9 dpi stained with hematoxylin and eosin (HE), or with green fluorescent protein (GFP) and MV N antibodies, respectively. Scale bar: 50 μm. Dpi: days post-inoculation.
(TIF)

S2 Fig Phenotype of MV-infected cells in experimentally infected NHP skin tissues collected at 9 dpi. (a–e) Split and merged multicolor fluorescent images of the insets shown in Fig 3A–3E. The phenotypes of MV-infected (green) cells in the dermis were (a) CD45^+^ leukocytes, (b) CD3^+^ T cells, (c) S100A8/A9 complex^+^ (MAC387) macrophages and (d) the cells surrounding CD31^+^ endothelial cells. In the epidermis, two types of MV-infected cells could be detected: (e) cytokeratin^+^ keratinocytes and cytokeratin^-^ cells (asterisk). Arrow indicates co-localization of GFP and specific cell marker. Dashed line indicates the basement membrane that separates the dermis (Dm) and the epidermis (Ep). Scale bar: 10 μm. Dpi: days post-inoculation.
(TIF)

S3 Fig Dynamics of MV infection and subsequent clearance in NHP skin tissues. Five high-power Z-stack focal infection sites in NHP skin tissues were chosen arbitrarily at high magnification. MV-infected cells were observed in different numbers in the (a) dermis and (b) epidermis at different time points. The cells in the dermis were hardly detectable at 13 dpi. In contrast, more MV-infected cells could still be detected in the epidermis at the same time point. The number of CD45^+^ leukocytes increased throughout the different time points in the (c) dermis and (d) epidermis. The number of CD45^+^ leukocytes increased in the dermis from 9 to 13 dpi, and in the epidermis between 11 and 13 dpi. Each symbol represents the number of cells counted in one infectious focus in one animal. Dpi: days post-inoculation.
(TIF)

S4 Fig Interaction between MV-infected cells and dermal cells in experimentally infected NHP skin tissues. (a–c) Representative split and merged multicolor fluorescent images shown in Fig 4. (a) An MV-infected CD3^+^ T cell (speckled green; arrow) was present in reticular dermis at 13 dpi, in close proximity to uninfected T cells (red). Merged image is shown in Fig 4B. (b) Close interaction between an MV-infected cell (green) with an HLA-DR^+^ APC (red), forming a long EGFP^+^ dendrite (arrow). Merged image is shown in Fig 4C. (c) MV-infected CD31^+^ endothelial cells (red; arrows) in close proximity to other MV-infected cells (green). Merged image is shown in Fig 4E. (d) Close interaction between an S100A8/A9 complex^+^ (MAC387) macrophage (red) and an MV-infected cell (green) in the dermis. Scale bar: 10 μm. Dpi: days post-inoculation.
(TIF)

S5 Fig Interaction between MV-infected cells and epidermal cells in experimentally infected NHP skin tissues. (a–c) Representative split and merged multicolor fluorescent images shown in Fig 4. (a–b) Sequential slides of MV-infected NHP skin at 13 dpi. (a) An MV-infected CD45^+^ white blood cell (arrow) in the basal epidermis. (b) This cell was negative for cytokeratin marker (arrow) and in close proximity to infected keratinocytes (green). (c) MV-infected keratinocytes in the absence of other infected cells in the observed two-dimensional plane. (d–e) Sequential slides of MV-infected NHP skin at 11 dpi. (d) Infiltrating CD45^+^ leukocytes (red) could be observed in the epidermis. (e) Many of these cells were S100A8/A9 complex^+^ (MAC387) macrophages (red). Arrows in (d) and (e) indicated one of the CD45^+^ S100A8/A9 complex^+^ macrophages in the epidermis at 11 dpi. Dashed line indicates the basement membrane that separates the dermis (Dm) and the epidermis (Ep). Scale bars of (a–c): 10 μm. Scale bars of (d–e): 50 μm. Dpi: days post-inoculation.
(TIF)

S6 Fig MV-infected LCs were not observed after *ex vivo* infection of human epidermal sheets. LCs (magenta) were present in abundance in human epidermal sheets. MV-infected cells (green) appeared at 2 dpi and their number increased by 4 dpi. However, none of these infected cells were LCs. Magenta: CD1a; Green: GFP; Blue: DAPI. Scale bar: 200 μm. Dpi: days post-inoculation.
(TIF)

S7 Fig Differentiated human primary keratinocytes expressed higher levels of nectin-4 than proliferating keratinocytes. The expression level of nectin-4 increased during differentiation. NCI-H358 and BLCL were included as positive and negative controls of nectin-4 expression, respectively.
(TIF)

S8 Fig Nectin-4 expression and cell-free virus production of human primary proliferating and differentiated keratinocytes from an EDSS1 patient. Despite the low nectin-4 expression in both proliferating and differentiated EDSS1 keratinocytes, the cells were susceptible to MV infection. Infection also resulted in production of infectious cell-free virus progenies. KS: rMV^KS^Venus(3); KS-N4b: rMV^KS-N4b^EGFP(3). EDSS1: ectodermal dysplasia-syndactyly syndrome.
(TIF)

S9 Fig Flow cytometry analyses of MV-infected emigrant cells from supernatants of *ex vivo* human skin cultures. Gating strategy to determine the percentages of MV-infected emigrant cells in supernatants of *ex vivo* human epidermis sheets, dermis sheets or full skin tissues. Autofluorescent cells were not included in the MV gate. The same gating strategy was applied throughout the experiments to all samples collected at 2, 4 and 7 dpi.
(TIF)
