# Supplementary figures and images for: Measles skin rash: Infection of lymphoid and myeloid cells in the dermis precedes viral dissemination to the epidermis
Source: PLoS Pathog. 2020 Oct 8;16(10):e1008253. doi: 10.1371/journal.ppat.1008253 (PMC7575069; doi:10.1371/journal.ppat.1008253)

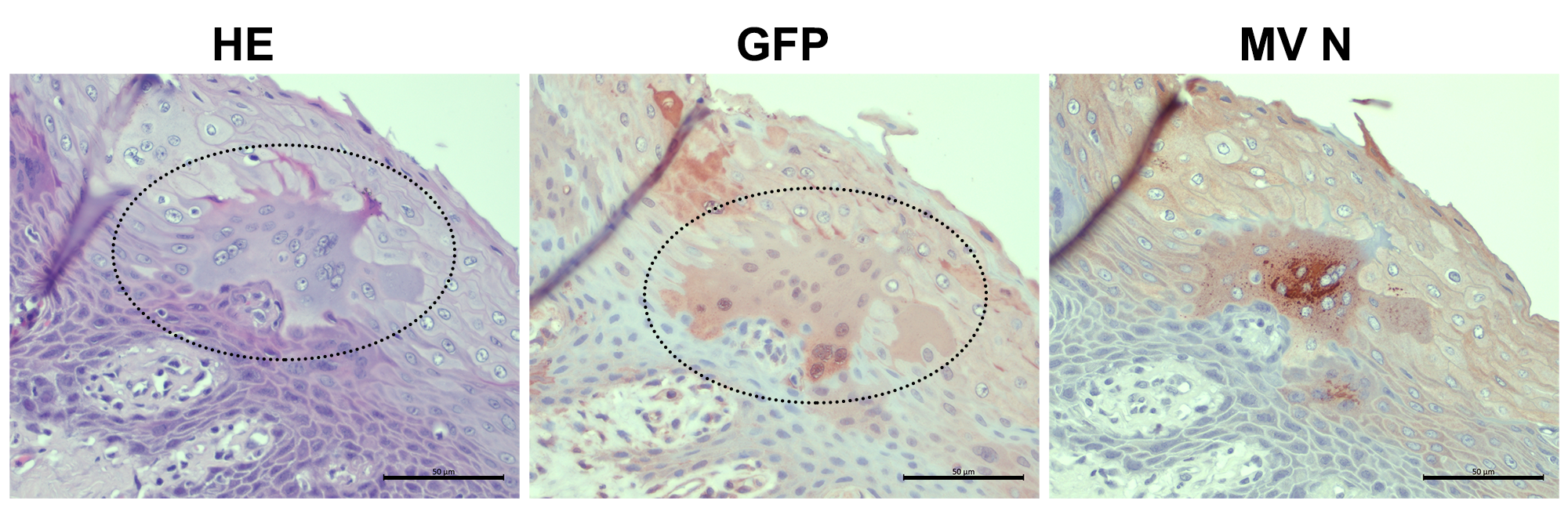

Supplement: S1 Fig — At 9 dpi, MV infection were mostly found in the dermis of experimentally infected NHPs. However, due to the focal nature of MV skin infection, MV-infected cells could sometimes also be detected in the epidermis. The progression of epidermal infection varied in different sites, ranging from only single-cell to multiple-cell infection. A syncytium (ellipse) was observable, albeit rarely, in the epidermis collected at 9 dpi stained with hematoxylin and eosin (HE), or with green fluorescent protein (GFP) and MV N antibodies, respectively. Scale bar: 50 μm. Dpi: days post-inoculation. (TIF) [file ppat.1008253.s001.tif]

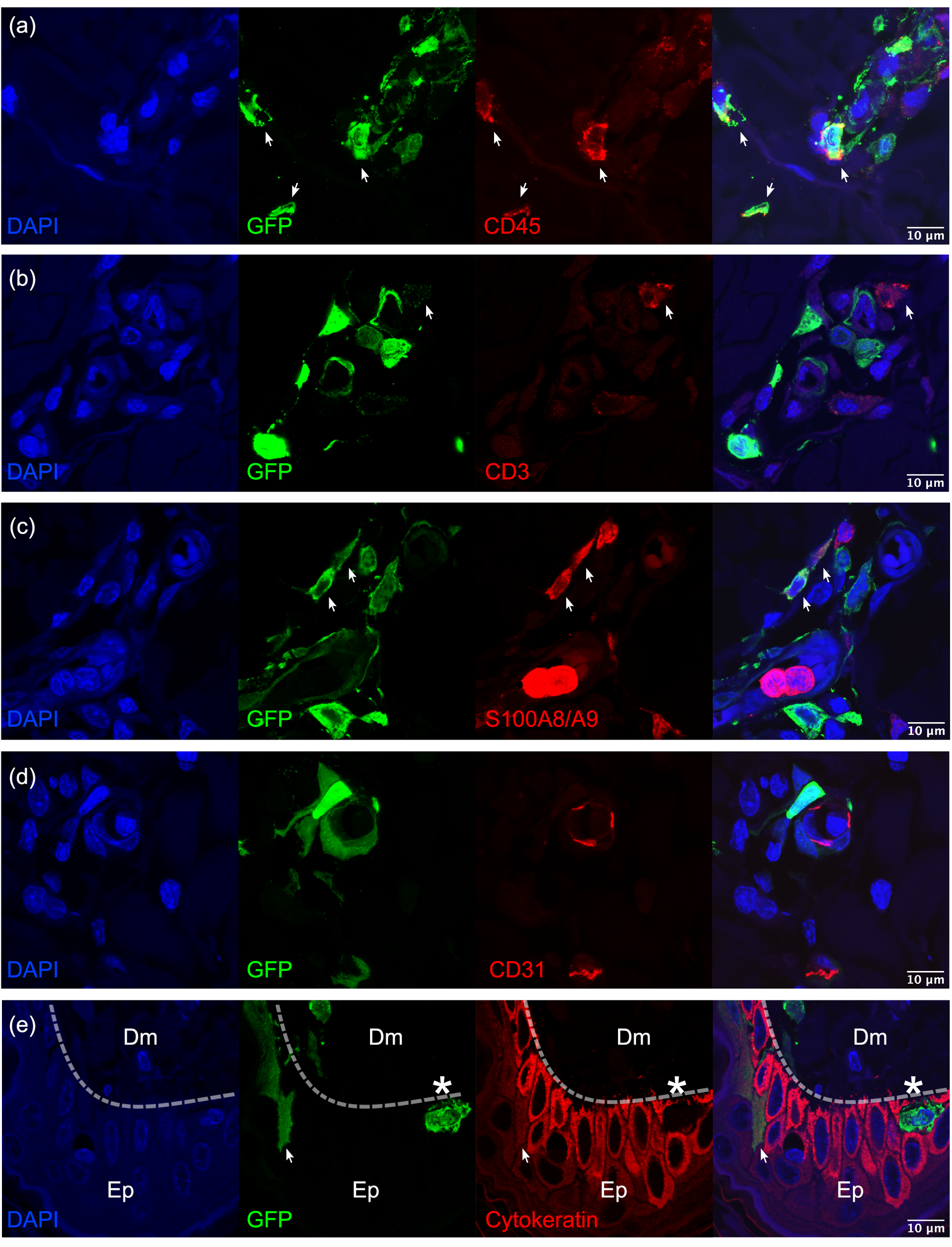

Supplement: S2 Fig — (a–e) Split and merged multicolor fluorescent images of the insets shown in Fig 3A–3E. The phenotypes of MV-infected (green) cells in the dermis were (a) CD45+ leukocytes, (b) CD3+ T cells, (c) S100A8/A9 complex+ (MAC387) macrophages and (d) the cells surrounding CD31+ endothelial cells. In the epidermis, two types of MV-infected cells could be detected: (e) cytokeratin+ keratinocytes and cytokeratin- cells (asterisk). Arrow indicates co-localization of GFP and specific cell marker. Dashed line indicates the basement membrane that separates the dermis (Dm) and the epidermis (Ep). Scale bar: 10 μm. Dpi: days post-inoculation. (TIF) [file ppat.1008253.s002.tif]

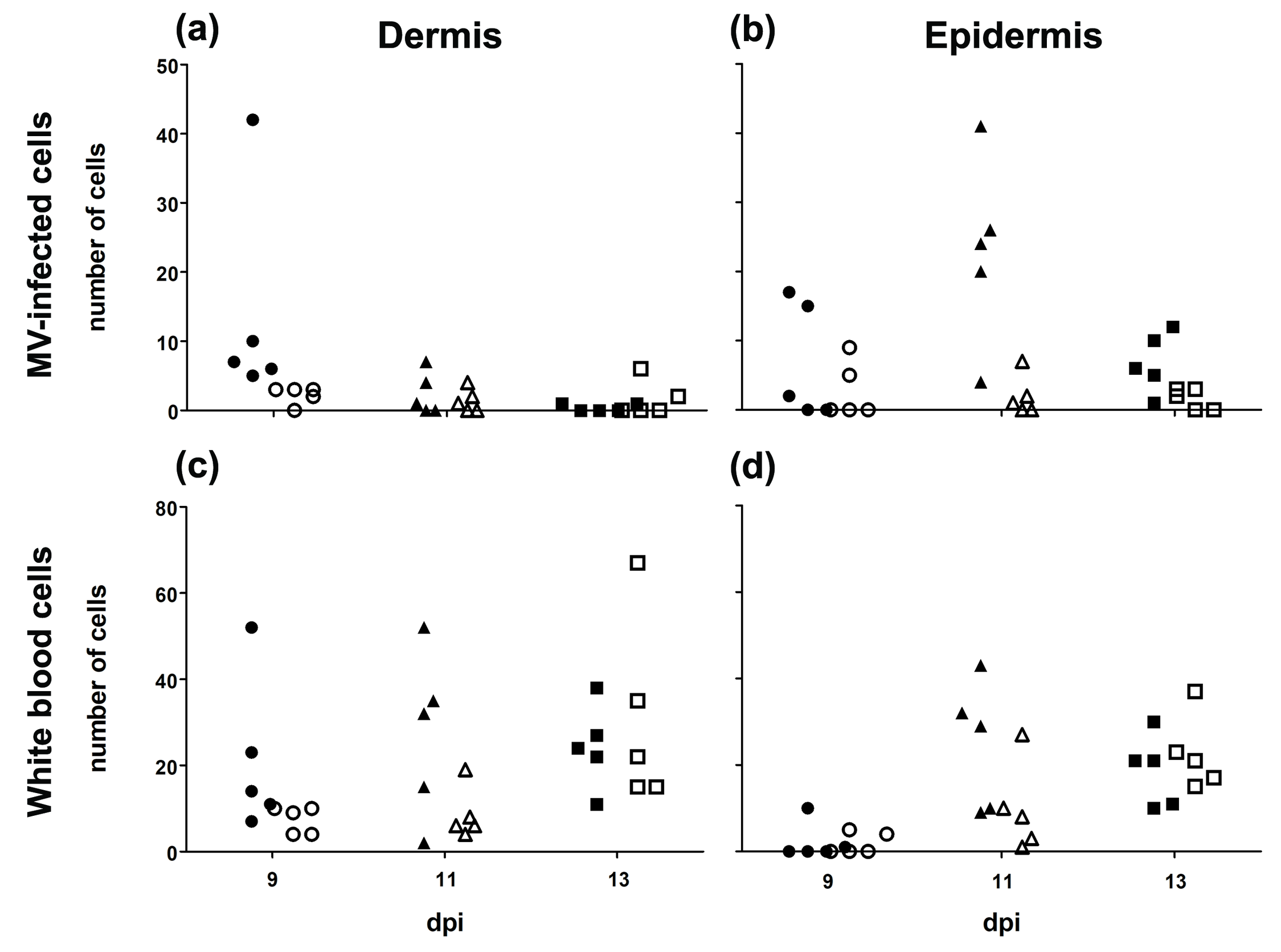

Supplement: S3 Fig — Five high-power Z-stack focal infection sites in NHP skin tissues were chosen arbitrarily at high magnification. MV-infected cells were observed in different numbers in the (a) dermis and (b) epidermis at different time points. The cells in the dermis were hardly detectable at 13 dpi. In contrast, more MV-infected cells could still be detected in the epidermis at the same time point. The number of CD45+ leukocytes increased throughout the different time points in the (c) dermis and (d) epidermis. The number of CD45+ leukocytes increased in the dermis from 9 to 13 dpi, and in the epidermis between 11 and 13 dpi. Each symbol represents the number of cells counted in one infectious focus in one animal. Dpi: days post-inoculation. (TIF) [file ppat.1008253.s003.tif]

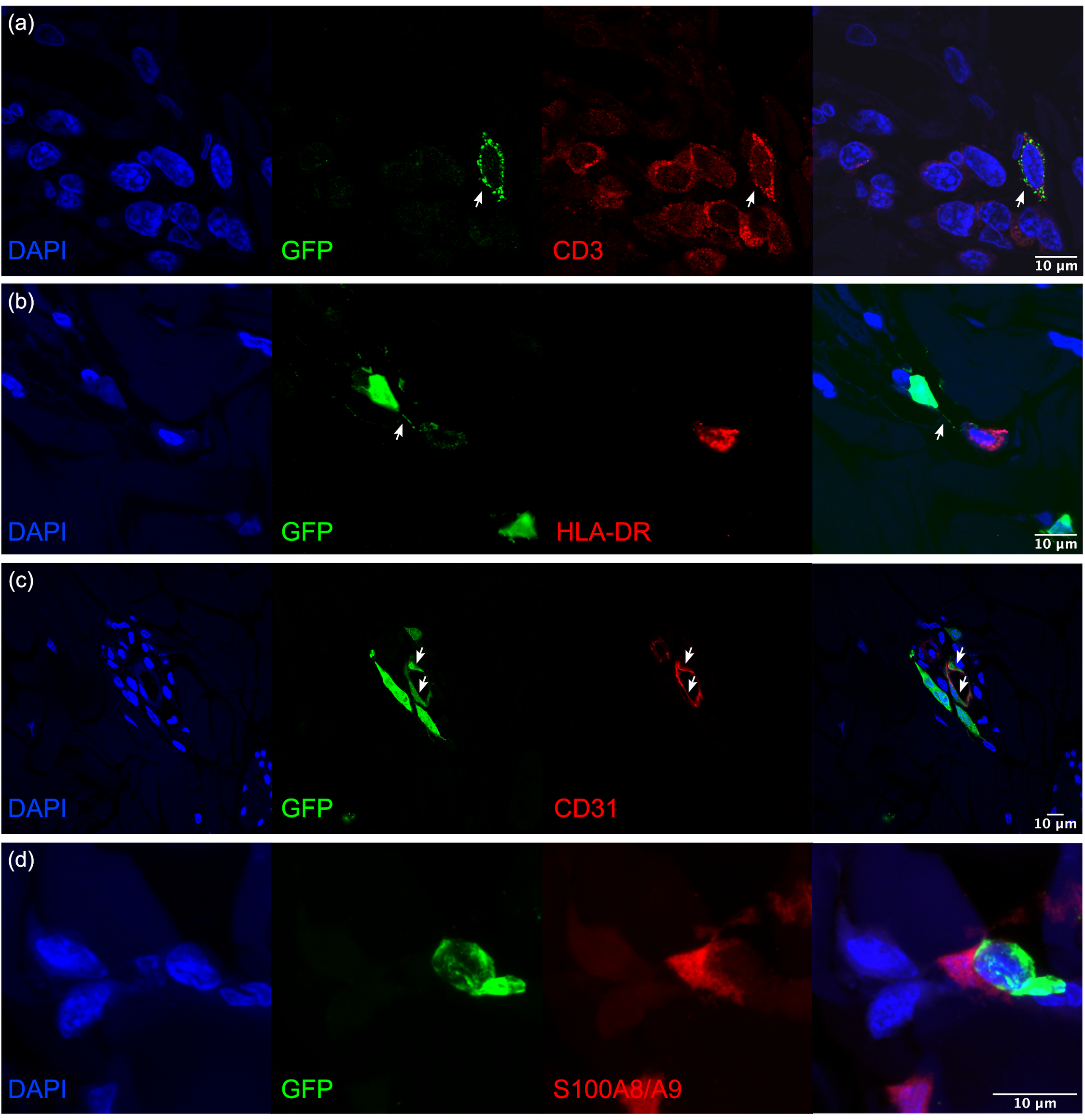

Supplement: S4 Fig — (a–c) Representative split and merged multicolor fluorescent images shown in Fig 4. (a) An MV-infected CD3+ T cell (speckled green; arrow) was present in reticular dermis at 13 dpi, in close proximity to uninfected T cells (red). Merged image is shown in Fig 4B. (b) Close interaction between an MV-infected cell (green) with an HLA-DR+ APC (red), forming a long EGFP+ dendrite (arrow). Merged image is shown in Fig 4C. (c) MV-infected CD31+ endothelial cells (red; arrows) in close proximity to other MV-infected cells (green). Merged image is shown in Fig 4E. (d) Close interaction between an S100A8/A9 complex+ (MAC387) macrophage (red) and an MV-infected cell (green) in the dermis. Scale bar: 10 μm. Dpi: days post-inoculation. (TIF) [file ppat.1008253.s004.tif]

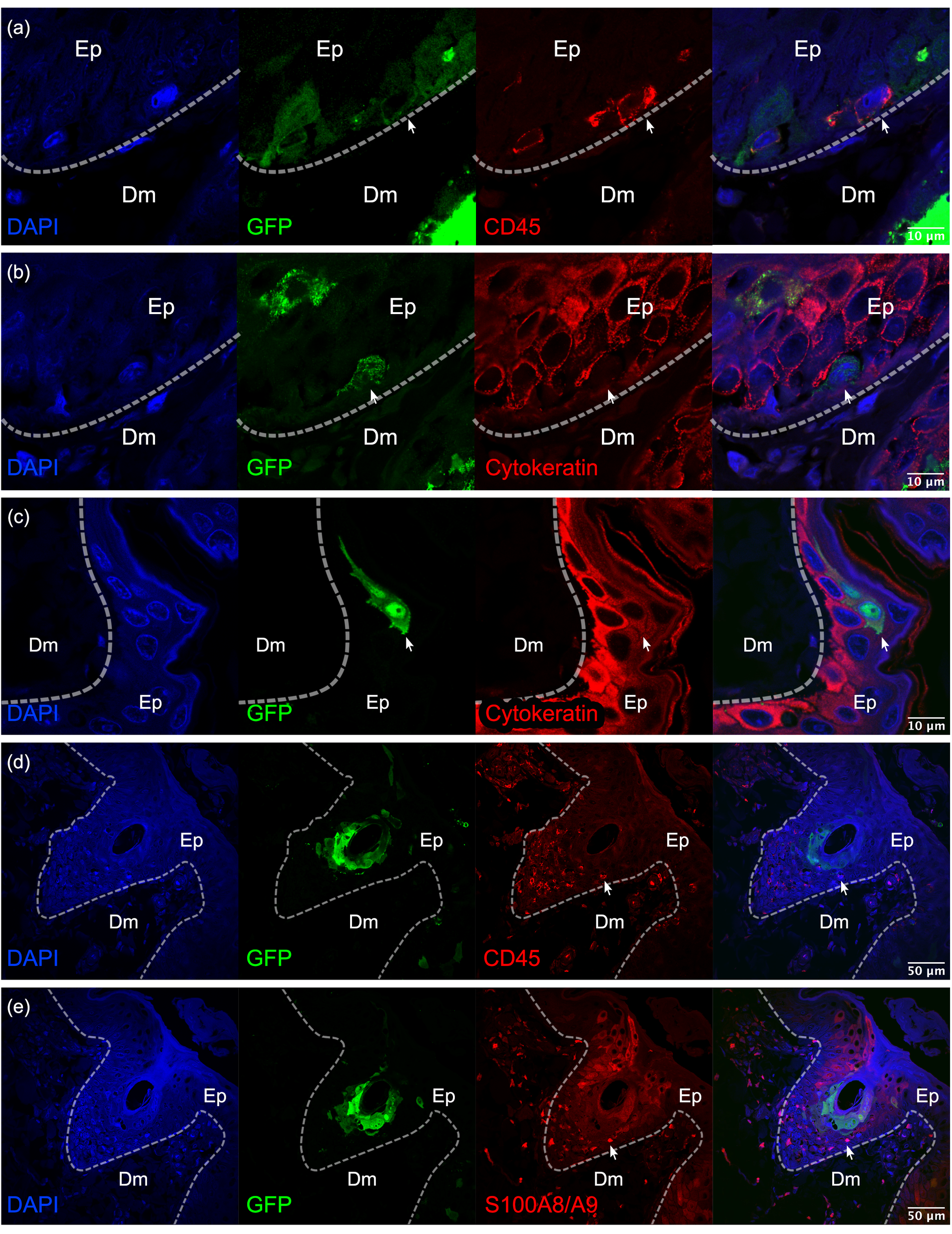

Supplement: S5 Fig — (a–c) Representative split and merged multicolor fluorescent images shown in Fig 4. (a–b) Sequential slides of MV-infected NHP skin at 13 dpi. (a) An MV-infected CD45+ white blood cell (arrow) in the basal epidermis. (b) This cell was negative for cytokeratin marker (arrow) and in close proximity to infected keratinocytes (green). (c) MV-infected keratinocytes in the absence of other infected cells in the observed two-dimensional plane. (d–e) Sequential slides of MV-infected NHP skin at 11 dpi. (d) Infiltrating CD45+ leukocytes (red) could be observed in the epidermis. (e) Many of these cells were S100A8/A9 complex+ (MAC387) macrophages (red). Arrows in (d) and (e) indicated one of the CD45+ S100A8/A9 complex+ macrophages in the epidermis at 11 dpi. Dashed line indicates the basement membrane that separates the dermis (Dm) and the epidermis (Ep). Scale bars of (a–c): 10 μm. Scale bars of (d–e): 50 μm. Dpi: days post-inoculation. (TIF) [file ppat.1008253.s005.tif]

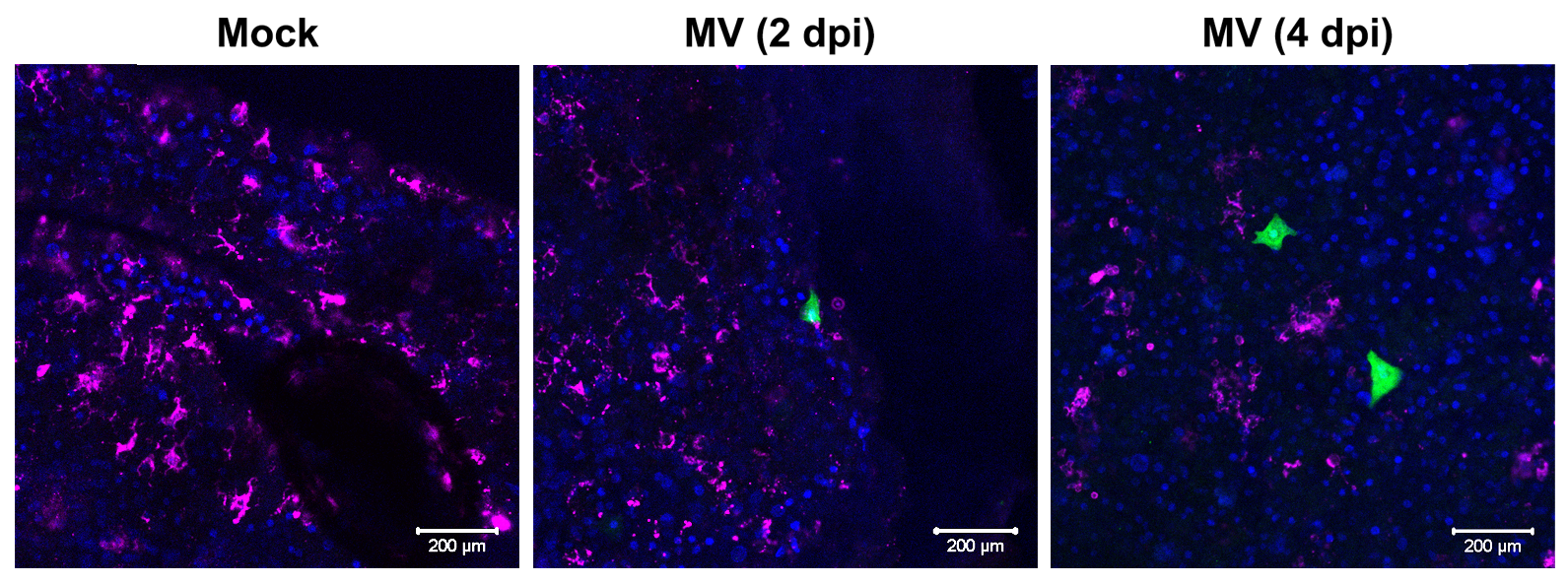

Supplement: S6 Fig — LCs (magenta) were present in abundance in human epidermal sheets. MV-infected cells (green) appeared at 2 dpi and their number increased by 4 dpi. However, none of these infected cells were LCs. Magenta: CD1a; Green: GFP; Blue: DAPI. Scale bar: 200 μm. Dpi: days post-inoculation. (TIF) [file ppat.1008253.s006.tif]

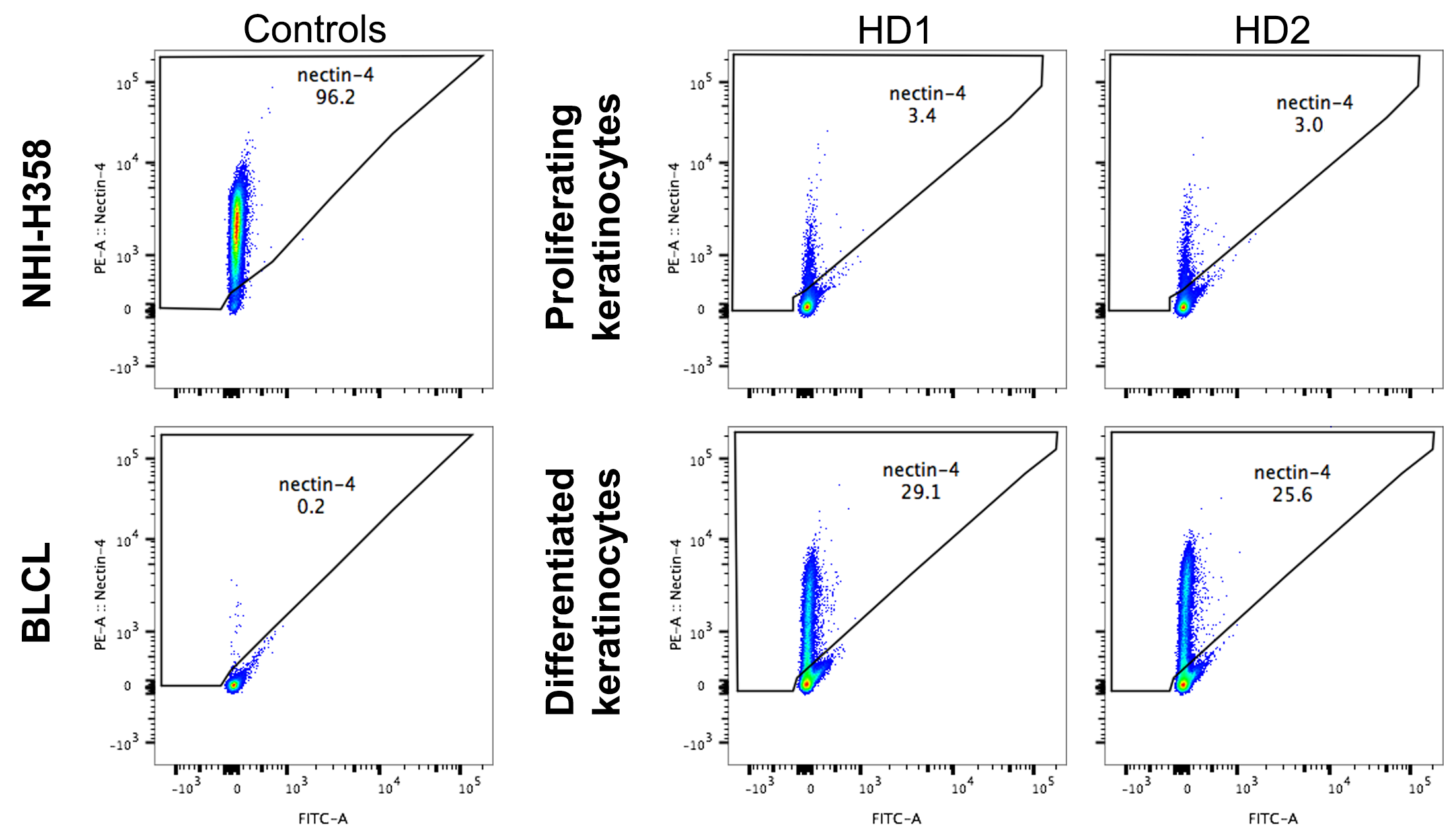

Supplement: S7 Fig — The expression level of nectin-4 increased during differentiation. NCI-H358 and BLCL were included as positive and negative controls of nectin-4 expression, respectively. (TIF) [file ppat.1008253.s007.tif]

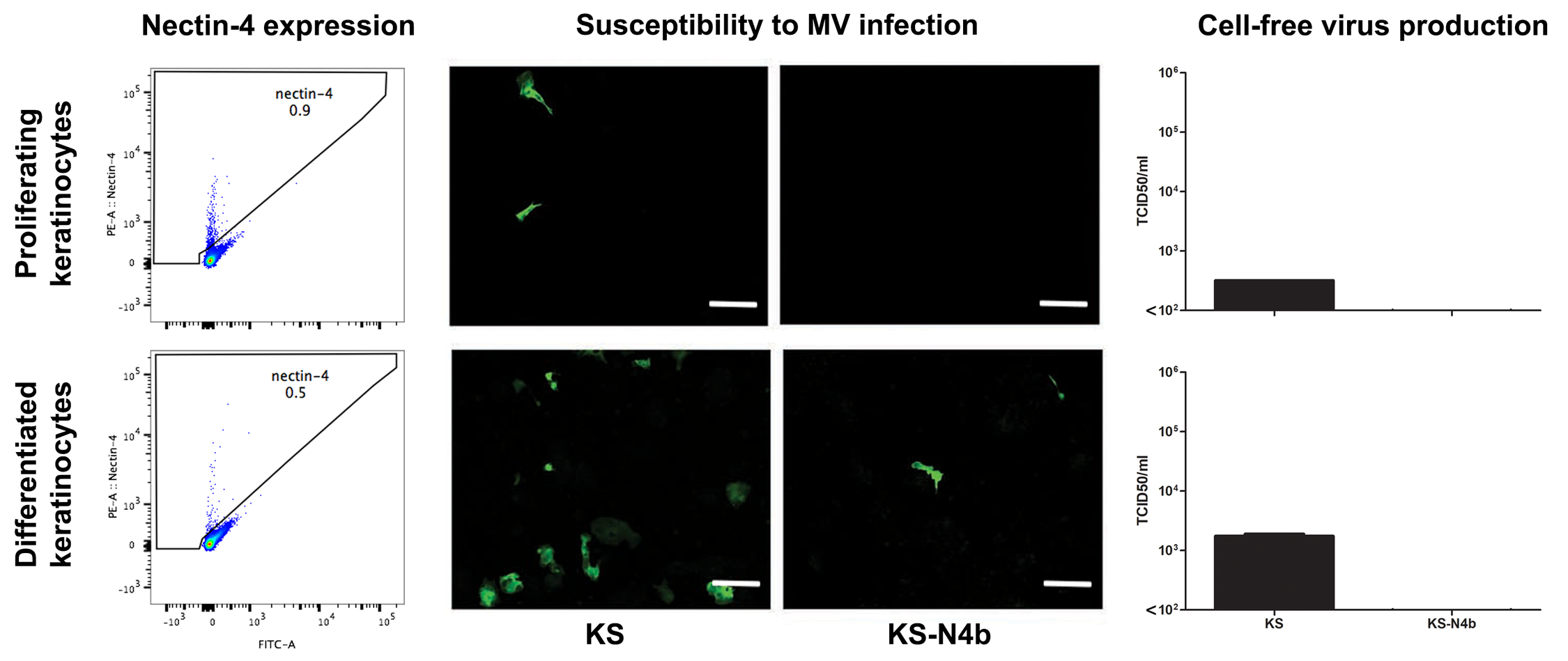

Supplement: S8 Fig — Despite the low nectin-4 expression in both proliferating and differentiated EDSS1 keratinocytes, the cells were susceptible to MV infection. Infection also resulted in production of infectious cell-free virus progenies. KS: rMVKSVenus(3); KS-N4b: rMVKS-N4bEGFP(3). EDSS1: ectodermal dysplasia-syndactyly syndrome. (TIF) [file ppat.1008253.s008.tif]

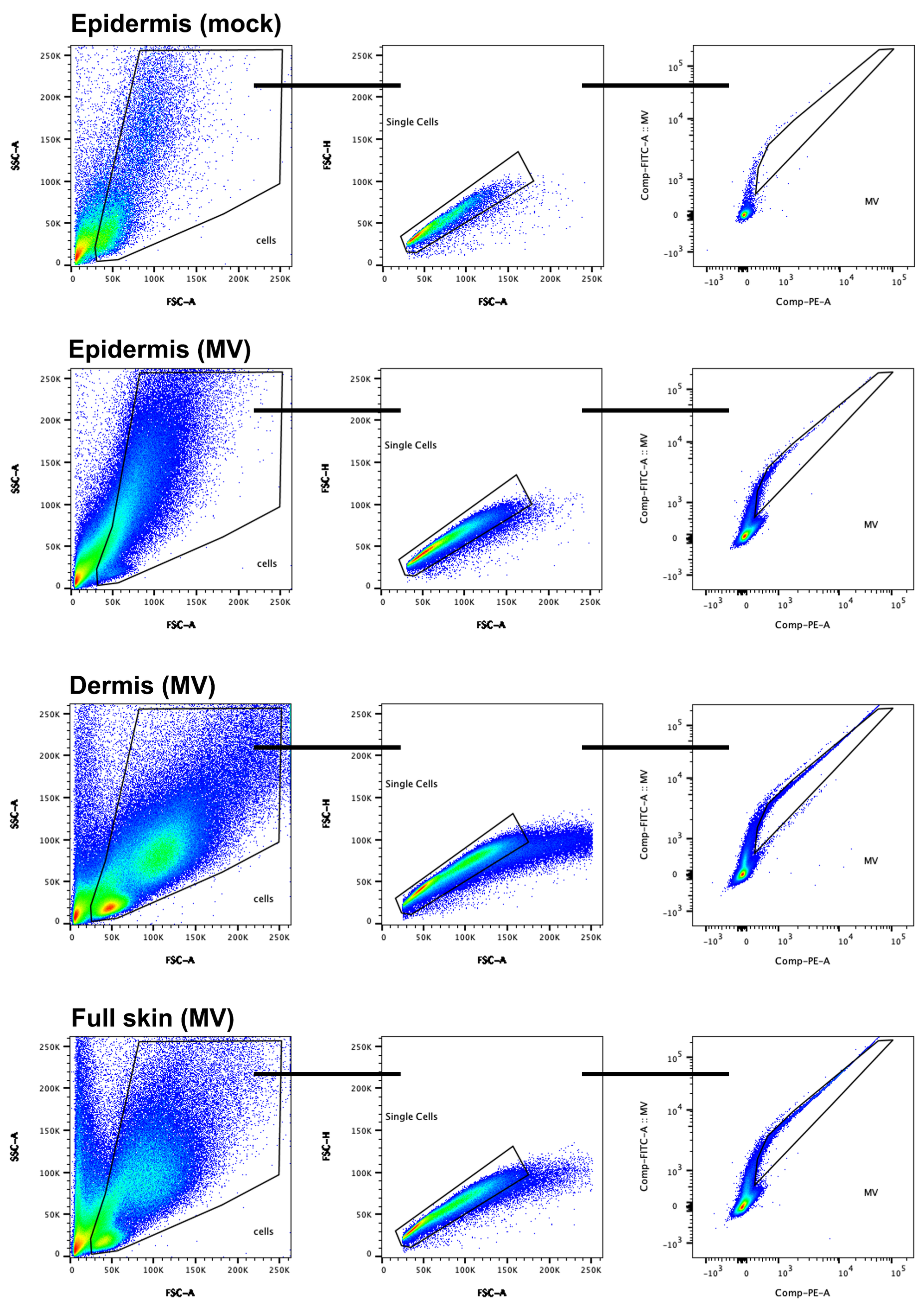

Supplement: S9 Fig — Gating strategy to determine the percentages of MV-infected emigrant cells in supernatants of ex vivo human epidermis sheets, dermis sheets or full skin tissues. Autofluorescent cells were not included in the MV gate. The same gating strategy was applied throughout the experiments to all samples collected at 2, 4 and 7 dpi. (TIF) [file ppat.1008253.s009.tif]
